# Rho/ROCK signaling and **α**-catenin mediate **β**-catenin–driven hyperplasia in the adrenal cortex via adherens junctions

**DOI:** 10.1172/JCI196271

**Published:** 2026-01-27

**Authors:** Mesut Berber, Betul Haykir, Nick A. Guagliardo, Vasileios Chortis, Kleiton Silva Borges, Paula Q. Barrett, Felix Beuschlein, Diana L. Carlone, David T. Breault

**Affiliations:** 1 Division of Endocrinology, Boston Children’s Hospital, Boston, Massachusetts, USA.; 2 Department of Pediatrics, Harvard Medical School, Boston, Massachusetts, USA.; 3 Department of Pharmacology, University of Virginia, Charlottesville, Virginia, USA.; 4 Department of Endocrinology, Diabetology and Clinical Nutrition, University Hospital Zurich and University of Zurich, Zurich, Switzerland.; 5 Medizinische Klinik und Poliklinik IV, Klinikum der Universität, Ludwig-Maximilians-Universität, Munich, Germany.; 6 The LOOP Zurich Medical Research Center, Zurich, Switzerland.; 7 Harvard Stem Cell Institute, Cambridge, Massachusetts, USA.

**Keywords:** Cardiology, Development, Endocrinology, Hypertension, Signal transduction, Tight junctions

## Abstract

How β-catenin (βCat) mediates tissue hyperplasia is poorly understood. To explore this, we employed the adrenal cortex as a model system given its stereotypical spatial organization and the important role βCat plays in homeostasis and disease. For example, excessive production of aldosterone by the adrenal cortex (primary aldosteronism [PA]) is a major cause of cardiovascular morbidity and is associated with βCat gain of function (βCat-GOF). Adherens junctions (AJs) connect the actin cytoskeletons of adjacent zona glomerulosa (zG) cells via a cadherin–βCat–α-catenin complex and mediate aldosterone production. Whether βCat-GOF drives zG hyperplasia, a key feature of PA, via AJs is unknown. Here, we showed that aldosterone secretagogues (K^+^ and AngII) and βCat-GOF mediated AJ formation via Rho/ROCK/actomyosin signaling. In addition, Rho/ROCK inhibition led to altered zG rosette morphology and decreased aldosterone production. Mice with zG-specific βCat-GOF demonstrated increased AJ formation and zG hyperplasia, which was blunted by Rho/ROCK inhibition and deletion of α-catenin (αCat). βCat also impacted AJ formation independently of its role as a transcription factor. Furthermore, analysis of human aldosterone-producing adenomas revealed high levels of βCat expression were associated with increased membranous expression of K-cadherin. Together, our findings identified Rho/ROCK signaling and αCat as key mediators of AJ formation and βCat-driven hyperplasia.

## Abstract 2

This study demonstrates that β-Catenin-driven hyperplasia in the adrenal cortex, a key feature of primary aldosteronism, is mediated through Rho-ROCK signaling and α-Catenin-dependent stabilization of adherens junctions, with significant implications for patients with aldosterone-producing adenomas.

## Graphical abstract

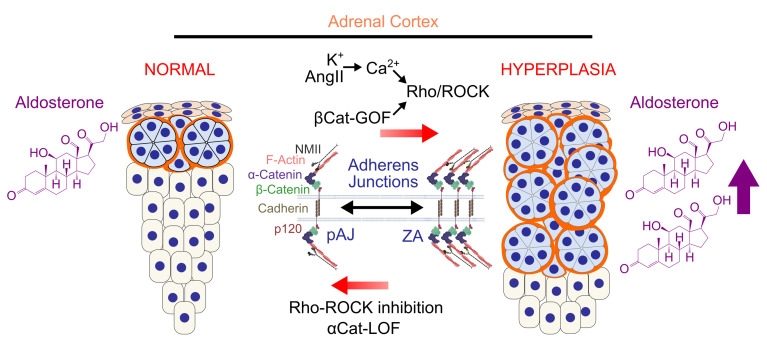

## Introduction

Primary aldosteronism (PA) is caused by aldosterone-producing micronodules (APMs) and adenomas (APAs) arising from zona glomerulosa (zG) cells of the adrenal cortex, which are characterized by autonomous overproduction of aldosterone (1). PA is the most common cause of secondary hypertension and includes 25% of patients with resistant hypertension (2). Delays in the diagnosis and treatment of PA result in a higher risk of cardiovascular morbidity, including stroke, heart failure, renal damage, and overall morbidity (3). While the majority of APMs and APAs arise from somatic mutations in ion channels associated with aldosterone production (1), the underlying cellular mechanisms driving hyperplasia within these lesions remain incompletely understood (4). Moreover, it is unclear to what extent these mechanisms intersect with pathways activated by plasma potassium (K^+^) or angiotensin II (AngII) signaling — the principal physiological regulators of zG size and aldosterone production (5).

Adherens junctions (AJs) are crucial mediators of cell–cell adhesion, structurally linking the actomyosin cytoskeleton of neighboring cells, and play a critical role in epithelial morphogenesis and tissue remodeling (6). AJs are composed of core structural elements, including transmembrane cadherins, β-catenin (βCat), and α-catenin (αCat). Among the core AJ components, αCat functions as an essential linker protein connecting the cadherin-βCat complex to the actomyosin cytoskeleton of the cell (7). AJs orchestrate rosette formation, a conserved developmental mechanism driving organogenesis and epithelial remodeling (8). Rosettes arise when 5 or more epithelial cells reorganize their cell–cell junctions into a flower-like structure. This process occurs through dynamic changes in AJs and actomyosin-driven cytoskeletal remodeling and facilitates organogenesis in a range of systems (9, 10). In the adrenal, the zG consists of laminin β1–encapsulated glomerular structures composed of multicellular rosettes (10), and in humans, expanded rosettes have been identified in aldosterone-producing cell clusters (11), a possible precursor to APAs.

The overall stability of AJs results from the dynamic balance between AJ assembly (formation) and disassembly, a process that is tightly regulated to allow cells to modulate their adhesion strength and respond to physiological cues during tissue homeostasis (12). Thus, dysregulation of AJ dynamics can dramatically impact tissue structure and function. For example, an increase in AJ formation is associated with the initiation of tumor formation, while a decrease in AJ formation is associated with epithelial-mesenchymal transition, which can ultimately result in tumor cell metastasis (13–15). Morphologically, AJs exist on a continuum between 2 distinct forms: punctate AJs (pAJs) and zonula adherens (ZAs) (6, 16, 17). pAJs are associated with transient weakening of AJs, enabling cell movement and tissue rearrangement, and consist of discrete points of cell adhesion. In contrast, ZAs result from increased AJ formation and stability, are essential for the maintenance of tissue integrity, and are composed of continuous and dense AJ clusters that span cell–cell boundaries. Various signaling pathways have been implicated in the regulation of AJ formation, including those downstream of Rho-GTPases (9, 18). For example, Rho-associated, coiled coil–containing kinase (ROCK) increases nonmuscle myosin II (NMII) motor activity through phosphorylation of the regulatory light chain of NMII (pMLC) (18). Moreover, the recruitment of NMII to the AJ and the motor activity of NMII are critical for the formation, expansion, and maintenance of ZAs (19). Thus, elucidating pathways that regulate AJs in the zG could constitute a meaningful advance in understanding the cellular mechanisms underlying zG hyperplasia.

While βCat functions as an AJ linker protein, it also serves as an essential transcription factor in the canonical WNT/βCat pathway, which is regulated, in part, via continuous βCat degradation (20). Stabilization of βCat, via phosphorylation or deletion of key degron domains, has been implicated in zG pathology in both humans and mice (21, 22). For example, somatic gain-of-function (GOF) mutations in *CTNNB1* (the gene encoding βCat) (βCat-GOF) result in increased aldosterone production (23, 24), and βCat stabilization is detected in more than 70% of APAs (24). Moreover, zG-specific βCat-GOF in mice leads to zG hyperplasia through a block in transdifferentiation of zG cells into zona fasciculata cells without a corresponding increase in proliferation (22, 24, 25). Despite the central importance of βCat-GOF in zG hyperplasia and APA formation, it remains unclear whether βCat-GOF’s effects are mediated through changes in gene expression or not.

Here, we investigated the role of Rho/ROCK signaling, αCat, and βCat-GOF as mediators of AJ formation and zG hyperplasia. We discovered that physiological stimulation with K^+^ and AngII enhanced AJ formation in the zG via Ca^2+^-induced Rho/ROCK signaling. In addition, βCat-GOF in the zG led to enhanced AJ formation, and disruption of AJs (via inhibition of Rho/ROCK signaling) blocked βCat-driven zG hyperplasia, underscoring a key role for AJ stability as a driver of zG hyperplasia. Consistent with this, deletion of αCat also disrupted zG morphology and blocked βCat-driven zG hyperplasia. βCat also impacted AJ formation independently of its role as a transcription factor. Finally, in human APAs, levels of βCat positively correlated with levels of K-cadherin (KCad), consistent with βCat being a driver of AJ stability, which has important implications for the development of targeted therapies for patients with PA.

## Results

### K^+^ and AngII stimulation enhance AJ formation via Rho/ROCK signaling.

**Figure 1:**
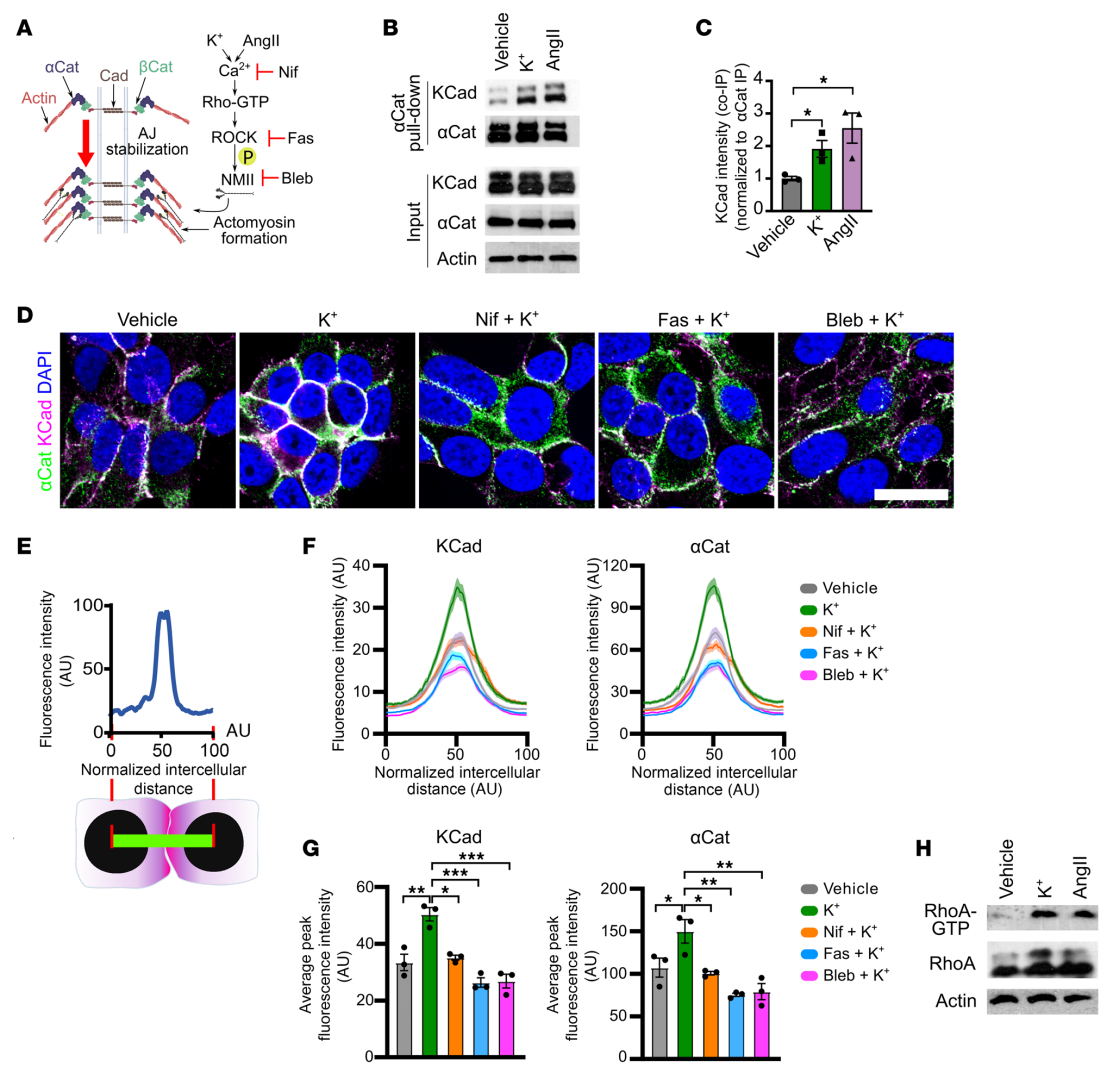
Aldosterone secretagogues increase AJ formation via the Rho/ROCK/NMII pathway. (**A**) Schematic illustrating AJ regulation by the K^+^/AngII-mediated Ca^2+^/ROCK/NMII pathway. Red arrow denotes AJ formation and stabilization. Nif, nifedipine; Fas, fasudil; Bleb, blebbistatin. (**B**) αCat co-IP in NCI-H295R cells treated with vehicle (11 mM NaCl), K^+^ (11 mM KCl), or AngII (100 nM) for 48 hours. KCad and αCat immunoblots. Input: whole-cell lysates. β-Actin: loading control. (**C**) Densitometric quantification of KCad/αCat co-IP (*n* = 3 independent experiments). (**D**) Representative image of αCat (green) and KCad (magenta) immunofluorescence in NCI-H295R cells stimulated with vehicle (11 mM NaCl), K^+^ (11 mM KCl) ± nifedipine (10 μM), fasudil (10 μM), or blebbistatin (10 μM) for 48 hours (1 hour preincubation with inhibitors or DMSO). Low-power images are in Supplemental Figure 2A. (**E**) Schematic of qLPA methodology. (**F**) qLPA of αCat and KCad fluorescence intensities represented in **D** presented as mean ± SEM (*n* = 90 cell–cell interfaces pooled from 3 independent experiments). (**G**) Average peak fluorescence intensity per experiment for KCad and αCat from **D** and **F** (*n* = 3). (**H**) RhoA activity in NCI-H295R cells treated with vehicle, K^+^, or AngII for 3 hours was assessed by pull-down assay. Immunoblots show active RhoA (RhoA-GTP) in precipitates (top), total RhoA in lysates (middle), and β-actin in lysates (bottom). Statistical significance determined by unpaired 2-tailed Student’s *t* test in **C** and 1-way ANOVA with Tukey’s multiple-comparison post test in **G**. *P < 0.05, **P < 0.01, ***P < 0.001. Data are presented as mean ± SEM. Scale bar: 20 μm.

To identify physiological mechanisms regulating zG function, we reanalyzed our phosphoproteomic dataset examining the effects of K^+^ stimulation on aldosterone production in the human NCI-H295R adrenocortical cell line (26). Gene Ontology and Reactome Pathway analyses revealed that K^+^ stimulation led to increased levels of phosphoproteins involved in cadherin binding, actin binding, and Rho-GTPase signaling, each of which is associated with an increase in AJ formation (Supplemental Figure 1A; supplemental material available online with this article; https://doi.org/10.1172/JCI196271DS1). To extend these findings, we assessed the effects of the aldosterone secretagogues K^+^ and AngII on AJs (including the transition from pAJs to ZAs) (Figure 1A and Supplemental Figure 1B). First, we used co-immunoprecipitation (co-IP) for αCat in NCI-H295R cells treated with either K^+^ or AngII. Both AngII and K^+^ significantly increased KCad association with αCat, suggesting increased AJ formation (Figure 1, B and C). To better characterize the K^+^-induced transition from pAJs to more stable ZAs, we developed a quantitative line profile analysis (qLPA) assay by adapting the fluorescence intensity line profiling techniques in ImageJ into a standardized quantitative approach, where the fluorescence intensity of KCad and αCat was measured along 50-pixel-wide lines drawn between adjacent nuclei and normalized to a 0–100 point scale. This method allowed for direct comparison of AJ proteins across experimental conditions. Consistent with the co-IP results, qLPA following stimulation with K^+^ revealed an increase in AJ proteins, suggesting a transition from pAJs to ZAs (Figure 1, D–G, Supplemental Figure 1C, and Supplemental Figure 2A). Similar results were observed following stimulation with AngII (Supplemental Figure 1D). Considering that both K^+^ and AngII converge on Ca^2+^ signaling, we next inhibited Ca^2+^ channels with nifedipine to determine the role of Ca^2+^ influx in AJ assembly. As predicted, nifedipine prevented K^+^-induced AJ enrichment (Figure 1, D, F, and G, and Supplemental Figure 2A). Together, these data indicate that aldosterone secretagogues led to an increase in AJ formation between zG cells through Ca^2+^-mediated signaling.

**Figure 2:**
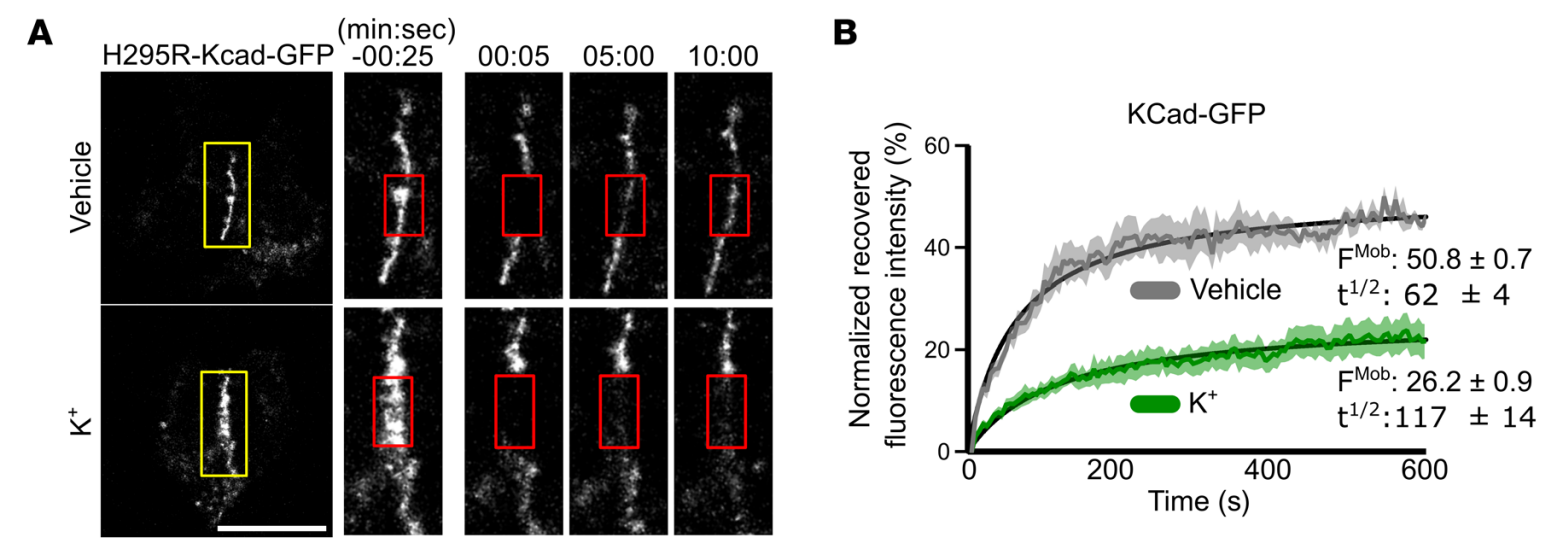
K^+^ increases AJ stability in NCI-H295R cells. (**A**) Representative FRAP images of KCad-GFP–expressing NCI-H295R cells. KCad-GFP at the cell–cell interface (yellow boxes) was photobleached (red boxes) at 00:00 and imaged over time. (**B**) Normalized KCad-GFP FRAP data from NCI-H295R cells treated with vehicle (11 mM NaCl) or K^+^ (11 mM KCl) for 48 hours (*n* = 5–7 cells pooled from 3 independent experiments). FMob, mobile fraction; *t_1/2_*, half-life recovery time (seconds). Data are presented as mean ± SEM. Scale bar: 20 μm.

Our phosphoproteomic analysis also identified Rho-GTPase signaling as a putative downstream mediator of K^+^ stimulation (Supplemental Figure 1A), indicating this pathway may mediate the enhancement of AJ formation. To confirm this, we treated NCI-H295R cells with K^+^ or AngII, which revealed increased RhoA-GTP activation (Figure 1H). Next, we assessed actomyosin formation, a downstream marker of RhoA activity (Figure 1A) (27), by staining for pMLC2 (28), F-actin, and NMIIB. K^+^ stimulation promoted MLC2 phosphorylation and robust actin polymerization at cell–cell contacts (Supplemental Figure 2, B and C), which directly correlated with increased fluorescence intensity and more continuous distribution of KCad and αCat along the normalized membrane interface. These changes reflect the molecular basis for the observed transition from pAJs to more stable ZAs, as assessed by qLPA (Supplemental Figure 2, D and E). Consistent with these findings, inhibition of ROCK activity (with fasudil) (29, 30) or myosin motor activity (with blebbistatin) (31) abrogated ZAs and actomyosin formation (Supplemental Figure 2, C–E). Indeed, inhibition of ROCK and NMII activity using fasudil, Y27632 (another ROCK inhibitor), and blebbistatin disrupted the membrane localization and junction formation of KCad and αCat, as evidenced by reduced fluorescence intensity at cell–cell interfaces (Figure 1, D, F, and G, and Supplemental Figure 2). Similar results were observed following stimulation with AngII (Supplemental Figure 2G). Finally, to establish whether the increase in AJ proteins seen following K^+^ stimulation was associated with an increase in AJ stability, we performed fluorescence recovery after photobleaching (FRAP) using a KCad-GFP fusion protein in NCI-H295R cells (Figure 2A). The results demonstrated that K^+^ stimulation markedly decreased the mobile fraction of KCad-GFP (thereby increasing the stable fraction), indicating that K^+^ stimulation led to increased AJ stability (Figure 2B). Taken together, these data indicate that aldosterone secretagogues promote AJ stability via Rho/ROCK/NMII signaling in adrenocortical cells via Ca^2+^ signaling.

### Rho/ROCK signaling mediates zG morphology and aldosterone production.

**Figure 3:**
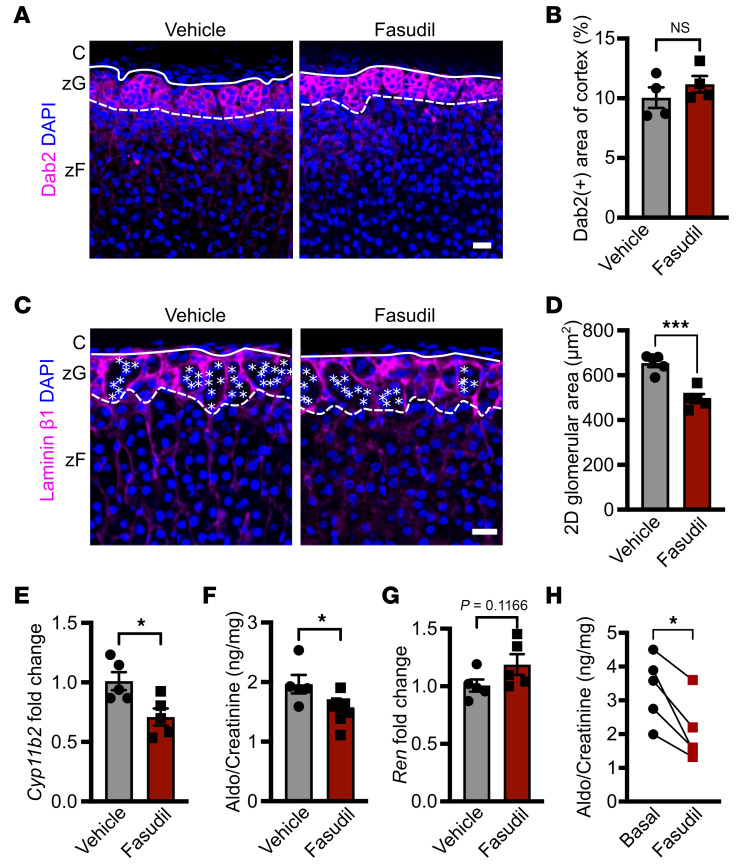
ROCK inhibition via fasudil decreases 2D glomerular area in the zG and blunts aldosterone production. (**A**) Representative images of Dab2 immunofluorescence in adrenal sections from 2-month-old control (*Cyp11b2^Cre/+^*) female mice treated with vehicle (PBS) or fasudil (30 mg/kg) every other day for 28 days. (**B**) Quantification of percentage Dab2^+^ area, as shown in **A** (*n* = 4, 4 mice). (**C**) Laminin β1 immunofluorescence in sections from 2-month-old female C57BL/6 mice treated as in **A**. Images highlight glomerular structures within the zG. (**D**) Quantification of 2D glomerular area via laminin β1 labeling represented in **C** (*n* = 5, 5 mice). (**E**) *Cyp11b2* mRNA expression in adrenals from vehicle- and fasudil-treated 2-month-old female C57BL/6 mice as in **A**, assessed by qPCR (*n* = 5, 5 mice). Data are presented as fold change relative to vehicle-treated controls normalized to *Gapdh* expression. (**F**) Measurement of 24-hour urinary aldosterone (Aldo) levels in 2-month-old female C57BL/6 mice treated as in **A**, assessed by RIA and normalized to creatinine (*n* = 5, 8 mice). (**G**) *Ren* mRNA expression in kidneys of 2-month-old female C57BL/6 mice treated as in **A**, assessed by qPCR (*n* = 5, 5 mice). Data are presented as fold change relative to vehicle-treated controls normalized to *18S* expression. (**H**) Measurement of 24-hour urinary aldosterone levels in 7- to 12-month-old control (*Cyp11b2^Cre/+^*) male mice before (basal) and after fasudil treatment (30 mg/kg) administered 6 times weekly for 14 days, assessed by RIA and normalized to creatinine (*n* = 5 mice). Statistical significance determined by unpaired 2-tailed Student’s *t* test or ratio paired *t* test in **H**. *P < 0.05, ***P < 0.001. Solid lines mark boundary between capsule (C) and zG, and dashed lines mark boundary between zG and zona fasciculata (zF). Each white asterisk denotes an individual cell within a zG rosette. Data are presented as mean ± SEM. Scale bars: 20 μm.

Given AJs are crucial for normal zG morphology (including zG rosettes) (10), we next assessed whether Rho/ROCK signaling mediated this process. We treated 2-month-old C57BL/6J WT female mice with fasudil for 4 weeks and assessed zG structure, gene expression, and aldosterone production. Compared with vehicle-treated mice, fasudil treatment led to a significant reduction in 2D glomerular area (a measure of zG morphology assessed using immunostaining for laminin β1) and zG rosettes, despite no change in the overall size of the zG (assessed using immunostaining for Dab2) (Figure 3, A–D). Next, we assessed the functional impact of fasudil on the zG, which revealed a decrease in *Cyp11b2* (encoding aldosterone synthase [AS]) expression (Figure 3E), and reduced aldosterone levels (Figure 3F), with a trend toward increased *Renin* (*Ren*) expression (Figure 3G). To confirm the reduction in aldosterone levels, we repeated the analysis with a more rigorous fasudil treatment protocol, incorporating pre- and posttreatment aldosterone measurements, which verified decreased aldosterone production following fasudil treatment (Figure 3H). Taken together, these results indicate a direct impact of fasudil on adrenal function and zG morphology. The modest changes in renal *Ren* mRNA levels may reflect the complex, multilevel regulation of *Ren* expression and suggest that both direct effects on the adrenal and compensatory renal responses may contribute to the observed hormonal changes in this well-coordinated endocrine system.

### βCat stabilization promotes AJ formation.

**Figure 4:**
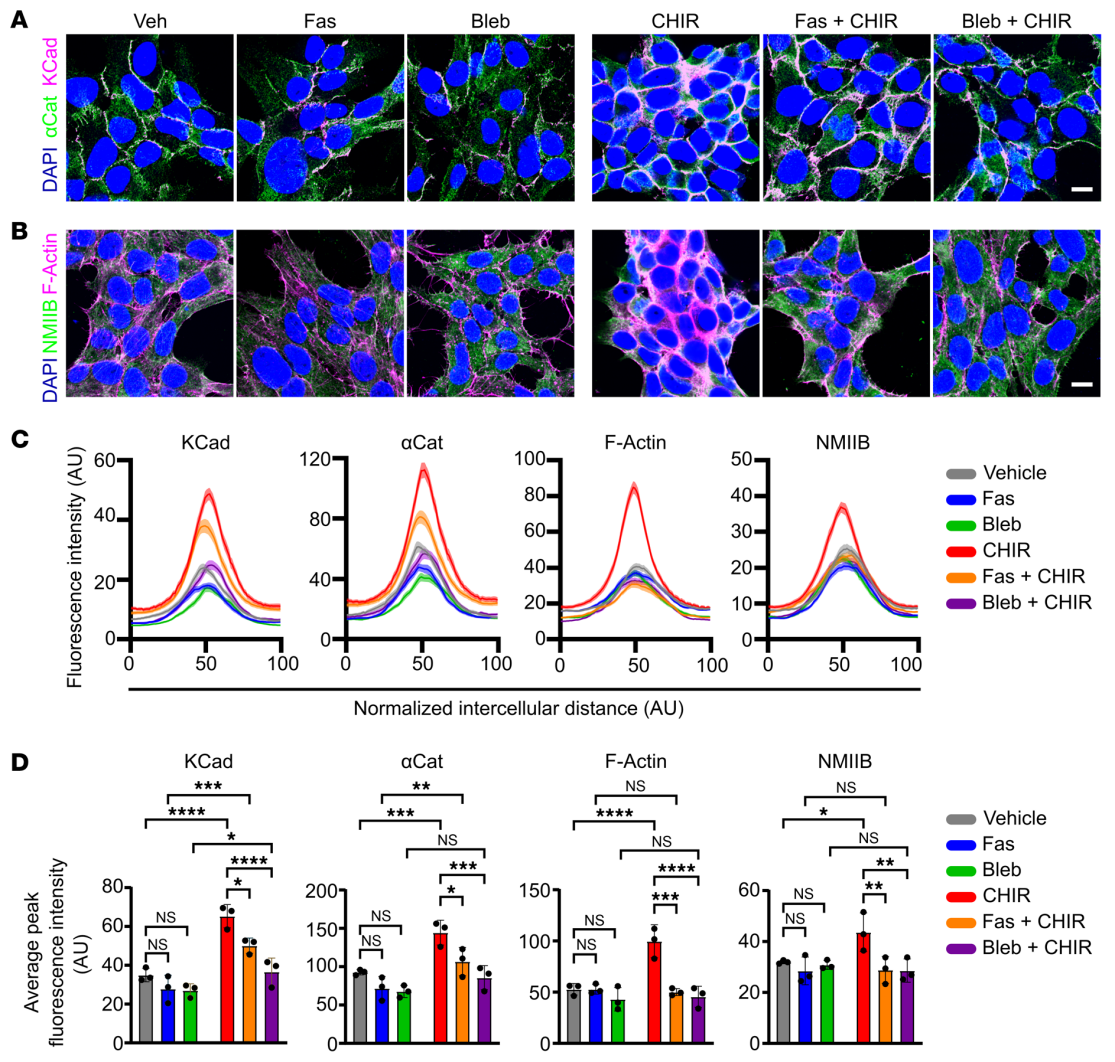
βCat stabilization via CHIR stimulation enhances AJ formation. (**A**) Representative images of αCat (green) and KCad (magenta) immunofluorescence in NCI-H295R cells treated with vehicle (Veh; DMSO), fasudil (Fas; 10 μM), or blebbistatin (Bleb; 10 μM) and stimulated with vehicle (DMSO) or CHIR 99021 (5 μM) for 48 hours (1 hour preincubation with inhibitors). (**B**) Representative images of NMIIB (green) immunofluorescence and F-actin (magenta) staining in NCI-H295R cells treated as in **A**. (**C**) QLPA of KCad, αCat, F-actin, and NMIIB fluorescence intensities from **A** and **B**. Lines represent mean ± SEM (*n* = 90 cell–cell interfaces pooled from 3 independent experiments). (**D**) Average peak fluorescence intensity for KCad, αCat, F-actin, and NMIIB per experiment, corresponding to data in **A**–**C** (*n* = 3). Data are presented as mean ± SEM. Statistical significance determined by 2-way ANOVA with Tukey’s multiple-comparison post test. *P < 0.05, **P < 0.01, ***P < 0.001, ****P < 0.0001. Scale bars: 20 μm.

While βCat is a core component of the AJ complex, recent evidence suggests that βCat can facilitate cadherin clustering (32), though whether increased levels of βCat can directly affect AJ formation has not been fully established. To explore this, we assessed the impact of βCat stabilization on AJ formation. First, we treated NCI-H295R cells with CHIR 99021 (CHIR), an inhibitor of GSK3β, to enhance βCat stabilization (Supplemental Figure 3, A–C). As expected, increased βCat stabilization led to an increase in βCat target gene (*LEF1* and *AXIN2*) expression (Supplemental Figure 3A). Treatment with CHIR also significantly enhanced membrane localization of KCad, αCat, and F-actin, leading to the formation of well-defined ZAs at cell–cell contacts, compared with pAJs in vehicle-treated cells (Figure 4, A–D, and Supplemental Figure 3, A–E), without affecting total KCad levels, as determined by immunoblot analysis (Supplemental Figure 3F), indicating that βCat likely promotes AJ formation through redistribution of existing proteins rather than through increased expression. To functionally assess whether CHIR-induced membrane localization of AJ components results in enhanced stability of cell–cell contacts, we performed a cell dissociation assay. Confluent monolayers of vehicle- or CHIR-treated NCI-H295R cells were detached and subjected to mechanical stress via repeated trituration. CHIR-treated cells showed significantly reduced dissociation compared with vehicle controls, remaining in larger intact fragments (Supplemental Figure 4, A and B), providing functional evidence that βCat stabilization promotes enhanced cell–cell contacts. Moreover, treatment with CHIR led to a pronounced aggregation of NCI-H295R cells into rosette-like clusters, reminiscent of the spatial organization observed in zG rosettes (Supplemental Figure 4C). Next, we assessed the impact of inhibiting the Rho/ROCK/NMII signaling pathway (using fasudil or blebbistatin) on CHIR-treated cells, which revealed a marked reduction in AJ formation and an attenuation of ZA formation (Figure 4, A–D). Similar results were observed following treatment with the ROCK inhibitor Y27632 (Supplemental Figure 3B). Next, we showed that CHIR increased pMLC2, a downstream marker of Rho/ROCK pathway activity (28), which was blocked by the addition of fasudil, providing a mechanistic link between βCat stabilization and Rho/ROCK signaling (Supplemental Figure 4, D and E). To validate these findings using a genetic model, we cultured primary zG cells isolated from control (*Cyp11b2^Cre/wt^:Ctnnb1^wt/wt^*) (33) and βCat-GOF mice (zG-specific *Cyp11b2^Cre/wt^:Ctnnb1^fl(Ex3)/wt^*) (22). Consistent with CHIR-treated NCI-H295R cells, primary zG cells from βCat-GOF adrenals demonstrated enhanced KCad staining at cell membranes compared with controls (Supplemental Figure 4F). Moreover, βCat-GOF primary zG cells exhibited increased aldosterone production compared with control cells (Supplemental Figure 4G), consistent with increased AJ formation leading to enhanced aldosterone production. Together, these findings demonstrate that the Rho/ROCK signaling axis is essential for βCat-mediated enhancement of AJ formation and multicellular organization in adrenocortical cells, providing mechanistic insight into how tissue architecture may be regulated in the zG.

**Figure 5:**
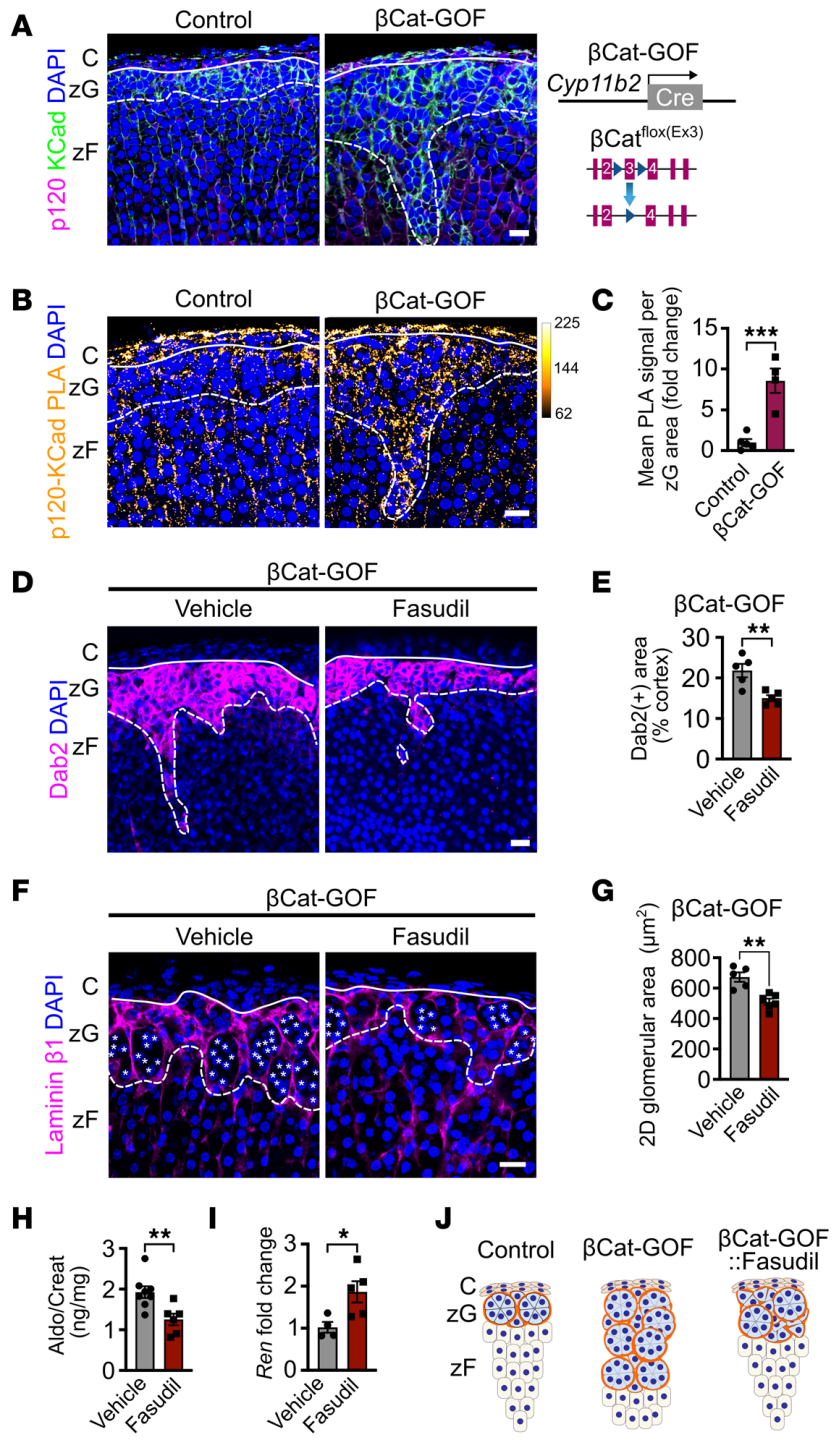
βCat stabilization strengthens AJs in the zG, while ROCK inhibition with fasudil prevents hyperplasia and reduces aldosterone production. (**A**) Representative images of KCad and p120-catenin (p120) immunofluorescence in adrenal sections from 2-month-old control and βCat-GOF female mice. Schematic of Cyp11b2^Cre/+^:Ctnnb1^fl(Ex3)/wt^ (βCat-GOF) mice. (**B**) Representative images of KCad/p120 PLA signal in sections from 2-month-old control and βCat-GOF female mice. (**C**) Quantification of PLA signal per zG area from control and βCat-GOF mice represented in **B** (*n* = 5, 4 mice). (**D**) Representative images of Dab2 immunofluorescence in sections from 2-month-old βCat-GOF female mice treated with either vehicle (PBS) or fasudil (30 mg/kg) administered every other day for 28 days. (**E**) Quantification of percentage Dab2^+^ area in the cortex, as shown in **D** (*n* = 5, 5 mice). (**F**) Representative images of laminin β1 immunofluorescence in sections from βCat-GOF female mice treated as in **D**. (**G**) Quantification of the 2D area of glomerular structures, as defined by laminin β1 labeling in **F** (*n* = 5, 6 mice). (**H**) Measurement of 24-hour urinary aldosterone (Aldo) levels of 4-month-old βCat-GOF female mice treated with either vehicle (PBS) or fasudil (30 mg/kg) administered 6 times per week for 28 days, assessed by RIA and normalized to creatinine (Creat) (*n* = 8, 6 mice). (**I**) *Ren* mRNA expression in kidneys of 4-month-old βCat-GOF female mice treated as in **H**, assessed by qPCR (*n* = 4, 5 mice). Data are presented as fold change relative to vehicle-treated controls normalized to *Gapdh* expression. (**J**) Schematic of control, βCat-GOF, and βCat-GOF:fasudil–treated mice. All statistical significance determined by unpaired 2-tailed Student’s *t* test. *P < 0.05, **P < 0.01, ***P < 0.001. Data are presented as mean ± SEM. Solid lines mark the boundary between the capsule (C) and zG, and dashed lines marks boundary between the zG and zona fasciculata (zF). Each white asterisk denotes an individual cell within a zG rosette. Scale bars: 20 μm.

To assess βCat’s impact on AJ formation in vivo, we employed adrenals from young adult (6–8 weeks of age) βCat-GOF and control mice. After confirming zG hyperplasia at this young age in βCat-GOF mice (22), as evidenced by an expanded Dab2^+^ zG (Supplemental Figure 5A), we performed immunostaining for KCad and p120 in the zG, which revealed a predominance of pAJs in the control zG and a marked increase in ZAs in the βCat-GOF zG (Figure 5A and Supplemental Figure 5B). We also measured *Cdh6* expression, which was comparable between βCat-GOF and control mice (Supplemental Figure 5C), suggesting βCat enhances KCad membrane localization primarily through spatial reorganization of existing protein pools (e.g., KCad) rather than via transcriptional upregulation, as with NCI-295R cells (Figure 4 and Supplemental Figures 3 and 4). To formally assess the binding of p120 to KCad — a proxy for AJ stability (34) — we next employed a proximity ligation assay (PLA) as a sensitive method for visualizing protein–protein interactions in situ (35), targeting KCad and p120. The analysis revealed a marked increase in the interaction of these AJ proteins in the zG of βCat-GOF mice compared with controls (Figure 5, B and C, and Supplemental Figure 5D). Together, these data indicate that βCat stabilization led to increased binding of AJ proteins, consistent with an increase in AJ stability.

To evaluate the functional relationship between Rho/ROCK signaling and βCat-driven zG hyperplasia, we administered fasudil or vehicle to 2-month-old female βCat-GOF mice. Fasudil treatment of βCat-GOF mice led to a marked reduction in Dab2^+^ zG area (Figure 5, D and E). In addition, fasudil treatment resulted in a marked reduction in zG glomerular area and zG rosettes (Figure 5, F and G). Next, given the progressive nature of the βCat-GOF phenotype with age (22), we confirmed these findings in both male and female mice at 4 months of age (Supplemental Figure 6, A–D). In addition, we showed treatment of βCat-GOF mice (with established zG cell hyperplasia) with fasudil led to decreased aldosterone production with a corresponding increase in *Ren* expression (Figure 5, H and I, and Supplemental Figure 6, E and F). To confirm these effects in aged βCat-GOF mice (22), which display more pronounced aldosterone elevation, we treated 1-year-old βCat-GOF male mice for 2 weeks with fasudil and measured aldosterone. Analysis of 24-hour urinary aldosterone excretion before and after treatment revealed fasudil treatment reduced levels to below those of littermate controls (Supplemental Figure 6G). Intriguingly, while fasudil treatment was able to block zG hyperplasia in βCat-GOF mice, it did not significantly affect expression of βCat’s transcriptional target genes *Lef1* and *Axin2* (Supplemental Figure 6H). Taken together, these findings establish that stabilization of βCat led to increased AJ formation via pMLC2 and that disruption of AJs through inhibition of Rho/ROCK signaling blunted βCat-GOF–driven hyperplasia and aldosterone production independent of significant changes in βCat-dependent target genes (Figure 5J).

Because βCat functions as both a transcription factor and a core AJ component, it is challenging to delineate the contribution of these 2 activities on AJ formation. To assess whether βCat’s transcriptional activity is required for AJ formation, we treated NCI-H295R cells with iCRT14 (a potent inhibitor of βCat transcription) (36). As expected, iCRT14 was able to block expression of canonical βCat target genes (*LEF1* and *AXIN2*) (Supplemental Figure 7A). Despite blocking βCat transcription activity, stabilization of βCat with CHIR was still able to promote KCad enrichment at cell–cell junctions (Supplemental Figure 7, B and C). This finding further supports that βCat’s role in AJ formation may be independent, at least in part, of its transcriptional activity.

### zG-specific αCat deletion impairs zG morphology.

To better assess the role of the AJ in zG and rosette formation, we next targeted αCat, an obligatory AJ adaptor protein that connects the cadherin-catenin complex to the actin cytoskeleton (Figure 1A) (7). We first generated zG-specific *Cyp11b2^Cre/wt^:Ctnna1^fl/fl^* mice, referred to as αCat-LOF (loss-of-function) mice. Analysis of 2-month-old αCat-LOF mice confirmed deletion of αCat in the zG compared with controls (Supplemental Figure 8A). We next assessed the impact of αCat-LOF on the zG, which revealed no change in the overall size or morphology of the zG, nor did it alter expression of the βCat target gene *Lef1*, compared with controls (Supplemental Figure 8, B–F). Despite this, αCat-LOF led to a significant reduction in zG 2D glomerular area and zG rosette number compared with controls (Supplemental Figure 8, G–I). These data indicate that despite no change in overall zG size between αCat-LOF and control mice, αCat is required for normal zG morphology, including formation of rosette structures.

Taken together, the above findings with iCRT14 and αCat-LOF mice decouple βCat’s nuclear and AJ functions and highlight a transcription-independent effect of βCat and the critical role of αCat. Despite these findings, we cannot exclude the possibility of nonjunctional roles for αCat, as has been described (37).

### zG-specific αCat deletion prevents βCat-GOF–driven zG hyperplasia.

**Figure 6:**
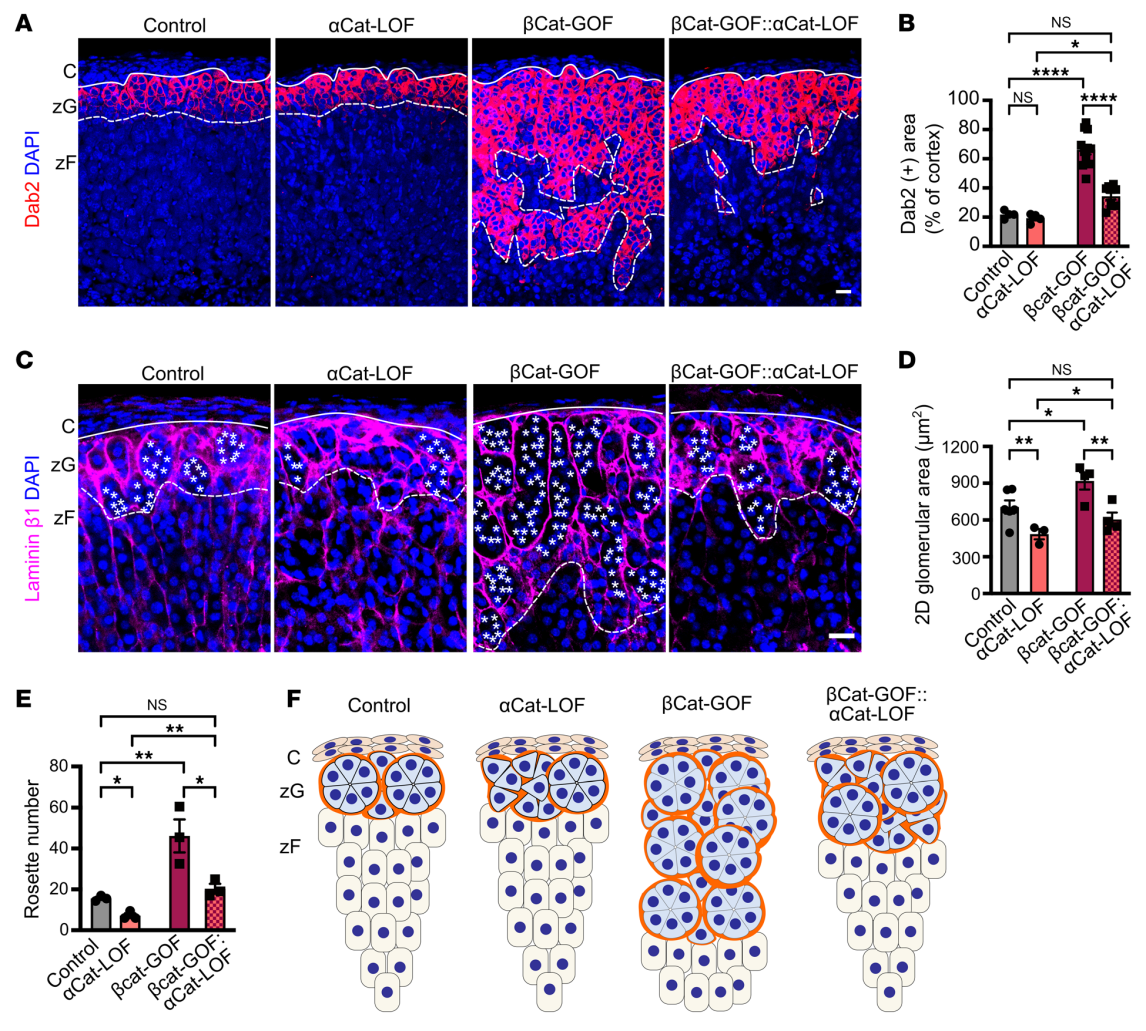
zG-specific αCat deletion attenuates βCat-GOF–induced zG hyperplasia and rosette expansion. (**A**) Representative images of Dab2 immunofluorescence in adrenal sections from 4-month-old control, αCat-LOF, βCat-GOF, and βCat-GOF:αCat-LOF male mice. (**B**) Quantification of percentage Dab2^+^ area in the cortex, represented in **A** (*n* = 4–12 mice). (**C**) Representative images of laminin β1 immunofluorescence in sections from 4-month-old control, βCat-GOF, αCat-LOF, and βCat-GOF:αCat-LOF male mice. (**D**) Quantification of the 2D area of glomerular structures, as defined by laminin β1 labeling in **C** (*n* = 3–6 mice). (**E**) Quantification of rosette number, defined as clusters of 5 or more cells within a single glomerular structure measured in regions of 500 × 500 μm, as shown in **C** (*n* = 3 mice per group). All statistical significance determined by 2-way ANOVA with Tukey’s multiple-comparison post test. *P < 0.05, **P < 0.01, ****P < 0.0001. Data are presented as mean ± SEM. Solid lines mark the boundary between the capsule (C) and zG, and dashed lines marks boundary between the zG and zona fasciculata (zF). Each white asterisk denotes an individual cell within a zG rosette. Scale bars: 20 μm.

To directly assess the role of αCat, and by extension AJs, in the context of βCat-driven zG hyperplasia, we generated zG-specific *Cyp11b2^Cre/wt^:Ctnnb1^fl(Ex3)/wt^:Ctnna1^fl/fl^* mice, referred to as βCat-GOF:αCat-LOF mice. We next compared 2D glomerular area and zG rosette number in control, βCat-GOF, αCat-LOF, and βCat-GOF:αCat-LOF mice. As expected, analysis of βCat-GOF mice revealed a 3-fold increase in Dab2^+^ zG area (66% ± 12% of the cortex) compared with control (22% ± 3%) and αCat-LOF (19% ± 3%) mice. Remarkably, analysis of βCat-GOF:αCat-LOF revealed a 50% decrease in Dab2^+^ zG area (34% ± 7% of the cortex) compared with βCat-GOF mice without affecting *Lef1* expression (Figure 6, A and B, and Supplemental Figure 9). Consistent with these results, we observed similar changes in 2D glomerular area and zG rosette number (Figure 6, C–E). These data indicate that αCat-dependent AJ formation is essential for βCat-driven zG hyperplasia (Figure 6F).

### Targeting AJs in βCat-GOF–driven zG hyperplasia leads to cell death.

To better understand the mechanism(s) responsible for the rescue of βCat-driven zG hyperplasia with fasudil and loss of αCat, we assessed cell proliferation (immunostaining for Ki67) and cell death (immunostaining for cleaved caspase-3) in the adrenals of βCat-GOF and βCat-GOF:αCat-LOF mice and in βCat-GOF mice treated with fasudil or vehicle. While the number of Ki67^+^ cells was comparable between genotypes and treatments, the number of cleaved caspase-3^+^ cells in the zG was significantly increased in βCat-GOF:αCat-LOF compared with βCat-GOF adrenals and in fasudil-treated βCat-GOF adrenals compared with vehicle (Supplemental Figure 10, A–H). Next, we treated NCI-H295R cells with different doses of fasudil (10, 50, and 100 μM) over 10 days, which led to a dose-dependent decrease in cell numbers, with the highest dose (100 μM) reducing cell numbers below starting levels (Supplemental Figure 10I). In addition, fasudil induced apoptosis in NCI-H295R cells at a dose of 100 μM, despite the presence of CHIR (Supplemental Figure 10J). Notably, fasudil-treated cells displayed increased segregation abnormalities, including intact and broken chromosome bridges and micronuclei — indicators of replication stress and aberrant mitosis (38) (Supplemental Figure 10K). These findings suggest that ROCK inhibition impairs faithful cell division, which may trigger checkpoint responses leading to apoptosis (38, 39). Beyond inducing apoptosis, qPCR analysis revealed that fasudil treatment significantly downregulated Cyclin D2 (*Ccnd2*) in vivo (Supplemental Figure 10L), a known βCat transcriptional target involved in G1/S transition (40, 41). Consistent with this, CHIR-induced upregulation of *CCND2* was completely blocked by fasudil cotreatment in NCI-H295R cells (Supplemental Figure 10M), indicating that ROCK activity is required for βCat-driven cell cycle progression. Together, these findings suggest that AJ destabilization suppresses hyperplasia through both impaired cell cycle progression and increased apoptotic cell death (39, 42–44).

### βCat expression correlates with increased AJs in APAs.

**Figure 7:**
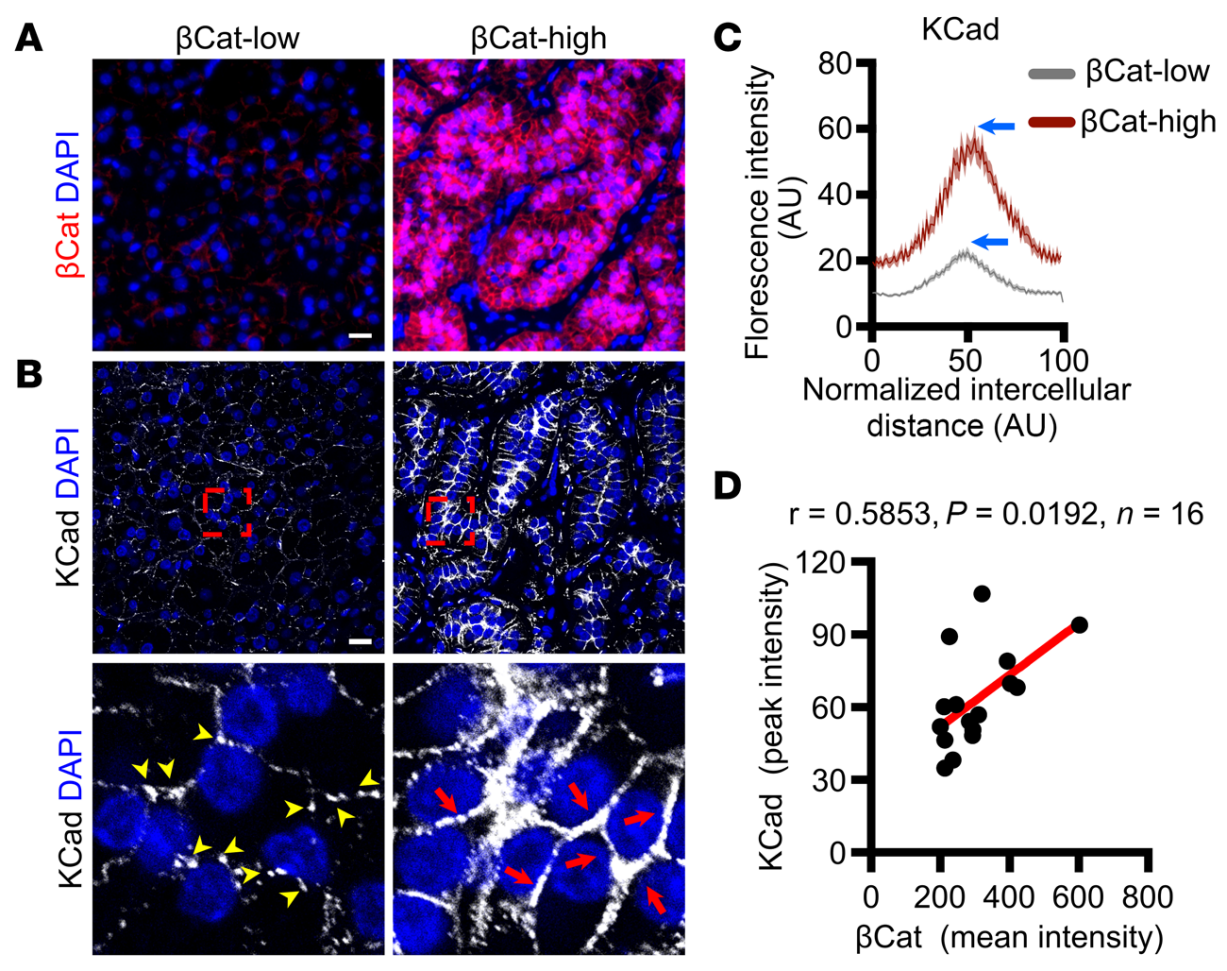
βCat expression positively correlates with KCad membrane localization in human APAs. (**A**) Representative images of βCat^lo^ and βCat^hi^ immunofluorescence in human APA sections. (**B**) Top panels, representative images of KCad immunofluorescence in βCat^lo^ (left) and βCat^hi^ (right) regions. Magnified views of regions marked by dashed red squares are shown in bottom panels. Yellow arrowheads denote pAJs, and red arrows denote ZAs. (**C**) QLPA of KCad fluorescence intensity in representative βCat^lo^ and βCat^hi^ regions, with blue arrows highlighting peaks of averaged KCad signal localized at approximately 50 cell–cell interfaces from randomly selected regions in APA sections. Lines represent mean and shaded areas represent standard error of the mean. (**D**) Spearman’s correlation analysis comparing βCat mean intensity and peak KCad intensity, as shown in **C**, from 16 human APA samples. The red line represents the regression fit (*r* = 0.5853, *P* < 0.0192). Scale bars: 20 μm.

Stabilization of βCat has been associated with a range of adrenal disorders, including APAs, where the majority of cases express high levels of βCat (24). To investigate whether high levels of βCat might contribute to an increase in AJs in human APAs, we analyzed paraffin-embedded sections from a cohort of 16 patients with APAs (Supplemental Table 1). We employed immunostaining for CYP11B2 (AS), βCat, and KCad. Focusing first on AS-expressing regions, in a blinded fashion, we randomly assessed βCat expression levels and independently scored AJ structures by determining the level of intercellular KCad (Figure 7, A and B, and Supplemental Figure 11A). While βCat expression levels showed no correlation with CYP11B2 expression in APAs, KCad peak intensity demonstrated a weak correlation (Supplemental Figure 11, B and C). Notably, APAs with higher βCat expression levels showed higher KCad levels at cell–cell contacts compared with those with lower βCat levels, which showed lower KCad levels (Figure 7, A–C). Using PLA as a proxy for AJ stability, we assessed the interaction between p120 and KCad, which revealed an increase in AJs in APAs with elevated βCat levels (Supplemental Figure 11D), mirroring the observations in βCat-GOF mice (Figures 5 and 6). To quantitatively and spatially assess the AJs, we scored approximately 50 cell pairs (across their cell–cell contacts) within each of 76 regions across all 16 APA samples for βCat levels (mean fluorescence intensity) and KCad levels (peak fluorescence intensity). Spearman’s correlation analysis comparing average data for each APA revealed a positive correlation between βCat and KCad levels (*r* = 0.58) (Figure 7D), with remarkable intratumor congruence (Supplemental Figure 11E). Together, these data suggest that elevated βCat expression is associated with enhanced AJ formation, consistent with a role for βCat stabilization in promoting epithelial cohesion and junctional integrity in APAs. In addition, these data highlight AJs as potential targets for the development of new treatments for these tumors.

## Discussion

Aberrant aldosterone production underlies PA, a condition affecting millions worldwide with substantial cardiovascular consequences. While the clinical importance of PA is well established, the molecular mechanisms governing zG expansion and hormone dysregulation remain incompletely understood. Our study addresses this knowledge gap by elucidating a signaling axis in the adrenal cortex that connects cell adhesion, tissue architecture, and hormone production. Specifically, we demonstrate that stabilization of βCat (βCat-GOF), beyond its recognized role as a transcription factor, drives AJ formation and subsequent zG morphogenesis. Our findings reveal that zG architecture, particularly the specialized rosette structures unique to the zG, depends on AJ integrity for both morphological maintenance and coordinated hormone secretion. Under normal conditions, we have uncovered that physiological aldosterone secretagogues (K^+^ and AngII) activate the Rho/ROCK/NMII signaling pathway leading to enhanced AJ stability. In addition, employing models of βCat-GOF, we discovered how this pathway is hijacked under pathophysiological conditions to drive zG hyperplasia and excess hormone production. Moreover, using zG-specific mouse models and pharmacological inhibitors of ROCK signaling, we showed how this pathway can be targeted to mitigate zG hyperplasia and excessive aldosterone production. In contrast to other adrenal states that demonstrate sexual dimorphism (26, 45–47), no meaningful differences were noted between male and female mice in this study regarding their morphological response to fasudil, αCat-LOF, βCat-GOF, or the combination of βCat-GOF and αCat-LOF. Together, these results underscore what appear to be fundamental regulatory mechanisms underlying adrenal homeostasis and the response to βCat-driven zG hyperplasia. Finally, the translational relevance of this work is underscored by our demonstration that βCat levels positively correlate with AJ formation in human APAs, further highlighting AJs as a potential therapeutic target for PA.

### Rho/ROCK signaling drives AJ formation in the adrenals.

AJs are highly dynamic structures whose formation and stability are precisely regulated by Rho signaling and actomyosin contractility (18, 48). We have previously shown that AJs in the zG mediate intercellular communication via rosette structures to coordinate dynamic aldosterone release in response to physiological stimulation (49). Yet, how such signals are transmitted from the extracellular space to the AJs remains poorly understood. Here, we extend our prior proteomic analysis of adrenal cells stimulated with K^+^, which identified increased levels of phosphoproteins regulated by Rho-GTPase activity (26). We demonstrate that stimulation of adrenal cells with K^+^ or AngII leads to robust activation of the Rho/ROCK/NMII pathway and formation of ZAs via Ca^2+^ signaling at cell–cell contact points. Underscoring the role of this pathway, inhibition of Rho/ROCK (using Y27632 or fasudil) or NMII (using blebbistatin) effectively prevents actomyosin contraction and disrupts ZA formation following stimulation. These findings provide compelling evidence that physiological stimuli enhance AJ stability through activation of Rho signaling, with additional kinases like myosin light chain kinase potentially contributing to this process. Consistent with this, human APAs have been identified with both increased abundance of RhoC (50) as well as missense mutations in Rho regulatory proteins (51, 52), supporting a model of autonomous pathological activation of this pathway. Collectively, our results establish a critical signaling axis through which aldosterone secretagogues trigger Rho activation in a physiological context, leading to enhanced AJ stability, cellular hyperplasia, and regulation of aldosterone secretion — a mechanism that may be hijacked in APAs and in adrenal hyperplasia.

### Disruption of AJs impairs zG rosettes and aldosterone production.

During mammalian development, rosettes are essential for the morphogenesis of a range of tissues, including the neural tube, kidney tubule, and pancreas, among others (8, 53). Rosettes primarily represent temporary developmental structures, which either resolve or open to form a lumen during mammalian development. Despite this, zG rosettes form during the first 6 weeks after birth in mice, are present throughout life, and notably do not form a lumen (10). In addition, we have previously shown that zG rosettes function as the fundamental unit of the zG and coordinate calcium-mediated electrical activity evoked by the aldosterone secretagogue AngII (49). Moreover, rosettes have also been described in the zG of adult humans, and larger rosettes have recently been associated with APMs (10, 11). To better understand the role of AJs in zG rosettes, we previously generated zG-specific βCat-LOF and βCat-GOF mice, which demonstrate marked disruption of rosettes in βCat-LOF adrenals and expansion of rosettes in βCat-GOF adrenals (10). However, βCat’s dual roles as a transcription factor and a structural component of the AJ (20) has limited our ability to fully delineate its impact on zG pathophysiology, particularly at the AJ. This duality also complicates efforts to isolate the specific contribution of AJs in this context. To address this challenge, we targeted αCat, a crucial AJ adaptor protein, by generating zG-specific αCat-LOF mice. Consistent with the requirement for AJs in rosette formation, αCat-LOF mice demonstrate a decrease in zG rosette numbers without impacting the overall size of the zG. Similarly, inhibition of Rho/ROCK signaling (with fasudil), in vivo, also decreased the size of zG glomerular structures. Taken together, these findings indicate that AJs, Rho signaling, and αCat are essential for normal formation of zG rosette structures in adult mammals. In addition, these results underscore the role of zG morphology, specifically the role of rosettes (rather than zG size per se), as being key to the level of aldosterone produced.

### βCat stabilization drives AJ formation.

Stabilization of βCat is reported in the majority of APAs (24), and mutations leading to βCat-GOF in mice drive zG hyperplasia (25, 54). βCat is localized in the nucleus, cytoplasm, and plasma membrane in zG cells (24) and is an essential component of the AJ. To better understand βCat’s role in AJ formation, we treated the adrenocortical cell line NCI-H295R with CHIR to induce βCat stabilization, which led to an increase in membrane localization of AJ proteins, including KCad, αCat, and F-actin, resulting in the formation of ZAs. Crucially, CHIR-mediated enrichment of KCad at the plasma membrane was not blocked by the βCat transcriptional inhibitor iCRT14, supporting a transcription-independent mechanism. Consistent with our work, Wnt-1–mediated stabilization of βCat has been shown to enhance cadherin-based adhesion by strengthening βCat binding to cadherins (55, 56). In addition, more recently, βCat condensation has been proposed to facilitate clustering of cadherin-βCat complexes and to promote nascent junction formation, further underscoring a direct role for βCat in regulating AJ formation (32).

Consistent with these observations, adrenals from βCat-GOF mice showed enhanced membrane labeling for KCad, despite no difference in *Cdh6* expression. Moreover, PLA revealed direct interactions between p120 and KCad, a proxy for AJ stability, in zG cells from βCat-GOF adrenals. Finally, we showed that in human APAs, βCat levels positively correlate with KCad levels and increased formation of AJs. Although these data from APAs are inherently correlative, they are reinforced by the causal evidence from our cell-based and mouse genetic models showing that βCat stabilization enhances AJ formation and multicellular organization. This integrative approach supports the conclusion that βCat plays a functional role in shaping tumor architecture in APA via modulation of cell–cell adhesion. Together, these data indicate that βCat stabilization drives enhanced AJ formation in mice and humans.

### βCat drives adrenal hyperplasia via enhanced AJ formation.

Constitutive activation of βCat-dependent Wnt signaling promotes tumorigenesis in multiple tissues, including the colon and the adrenal (25, 57). In addition, it is well known that nuclear βCat associates with TCF/LEF transcription factors to promote proliferation (57, 58). In contrast, we have previously shown that zG-specific βCat-GOF leads to zG hyperplasia without an increase in zG cell proliferation (22). To further explore this paradox, we assessed the role of AJs in zG-specific βCat-GOF mice using bigenic βCat-GOF:αCat-LOF mice. Consistent with our previous findings (22), βCat-GOF led to a progressive increase in zG hyperplasia. In contrast, morphological analysis of bigenic adrenals indicates that αCat, and by extension AJs, are required for βCat-driven zG hyperplasia. Interestingly, this requirement for αCat in βCat-driven hyperplasia mirrors findings from the Apc^Min^ mouse model of intestinal cancer, where the simultaneous loss of αCat and Apc during tumor initiation led to suppression of adenoma formation (59), suggesting a conserved role for αCat in supporting early stages of βCat-mediated tissue expansion across different organ systems.

### AJ disruption sensitizes hyperplastic zG cells to apoptosis.

The increased cleaved caspase-3 immunostaining observed in both βCat-GOF:αCat-LOF adrenals and fasudil-treated βCat-GOF mice aligns with established mechanisms whereby AJ disruption influences cell survival (42–44). Previous studies have demonstrated that disruption of cadherin-catenin complexes can sensitize epithelial cells to undergo apoptosis through multiple pathways, including exposing death receptors such as Fas to their ligands when AJ integrity is compromised (42), and through loss of the normal cellular microenvironment that supports cell survival (44). In the adrenals, zG cells are organized into specialized multicellular rosettes formed through AJ-mediated constriction. Disruption of this highly organized AJ-dependent architecture may be particularly effective at promoting apoptotic elimination of hyperplastic cells by destabilizing the structural integrity that normally supports cell survival, effectively counterbalancing the hyperplastic drive imposed by constitutive βCat activation. Beyond promoting apoptosis, ROCK inhibition may also impair cell cycle progression by downregulating the βCat target gene *Ccnd2*. Notably, while fasudil treatment did not significantly alter expression of canonical βCat target genes such as *LEF1* and *AXIN2*, *CCND2* (a well-established βCat transcriptional target) (40) was downregulated by ROCK inhibition both in vivo and in vitro. This selective effect on *CCND2* may reflect AJ-dependent modulation of βCat transcriptional activity or ROCK-dependent regulation of cell cycle progression independent of AJ destabilization, although the precise mechanism warrants further investigation. In addition, ROCK inhibition induces replication stress–associated chromosome segregation abnormalities. Consequently, ROCK inhibition restrains βCat-driven zG hyperplasia through multiple complementary mechanisms — impaired proliferation, replication stress, and enhanced apoptotic cell death — that together create multiple bottlenecks opposing zG expansion. While the complete molecular mechanisms linking AJ destabilization to activation of cleaved caspase-3 and suppression of *Ccnd2* in this rosette-based tissue warrant further investigation, our study highlights a critical role for AJs in the pathogenesis of βCat-driven zG hyperplasia in mice. Additional studies using mouse models of APAs are needed to define the functional significance of AJs in adenoma formation.

### Molecular heterogeneity and AJ regulation in human APAs.

The βCat-GOF mouse model provides a valuable platform for investigating how zG hyperplasia contributes to PA. In humans, however, PA typically originates from somatic mutation–driven focal hyperplasia rather than from global βCat activation or diffuse hyperplasia (60). Notably, aberrant βCat stabilization occurs in roughly 70% of APAs, even though direct *CTNNB1* mutations account for only 3%–5% of cases. By contrast, somatic alterations in ion channels and pumps — particularly *KCNJ5*, *CACNA1D*, and *ATP1A1* — are far more prevalent, with *KCNJ5* mutations alone comprising 38%–70% of APAs across different populations (61). Interestingly, APAs harboring *KCNJ5* mutations exhibit lower rates of βCat stabilization compared with other genotypes, highlighting distinct molecular pathways and suggesting potential subclassification within APAs. Moreover, some APAs, regardless of βCat status, harbor missense mutations in Rho-GTPase–activating proteins (51, 52), which inactivate Rho signaling, or display upregulated RhoC expression, particularly in *KCNJ5*-mutant tumors (50). Collectively, these findings underscore the complex interplay of signaling networks in APA pathogenesis.

Correlation analyses revealed distinct relationships between these molecular markers in our cohort of 16 human APAs. While βCat expression levels showed no significant correlation with CYP11B2 expression, KCad peak intensity demonstrated a weak but detectable correlation with CYP11B2. Importantly, βCat expression levels strongly correlated with intercellular KCad levels. Although the sample size is limited in this analysis, this differential correlation pattern supports the interpretation that βCat’s primary role in APAs is independent of the mechanisms directly regulating aldosterone production. Instead, βCat likely contributes to adenoma development through its role in stabilizing AJs and promoting epithelial cohesion, consistent with the 2-hit model of APA pathogenesis (2, 23, 62). The weak correlation between KCad and CYP11B2 may reflect their coordinated involvement in Ca^2+^-dependent signaling processes. We have previously shown that optimum aldosterone synthesis requires coordinated calcium bursts in response to secretagogues like AngII, which is mediated by AJs (49). Functionally, APAs with higher βCat expression levels showed significantly elevated KCad levels at cell–cell contacts compared with those with lower βCat levels. Furthermore, p120-catenin binding to KCad was enhanced in APAs with high βCat levels, indicating robust AJ stability in these tumors. p120-catenin plays a critical role in stabilizing cadherin-catenin complexes at the cell surface by preventing cadherin endocytosis and proteasomal degradation (34), thus providing a mechanistic basis for the observed correlation between βCat and AJ integrity. Collectively, this evidence highlights the critical involvement of AJ-associated pathways in human APA pathogenesis. The precise somatic mutation status of these tumor samples remains to be determined. Future studies leveraging expanded human APA cohorts with systematic mutational analyses are warranted.

### ROCK inhibition blocks zG hyperplasia and represents a potential therapeutic approach for PA.

ROCK inhibitors have demonstrated therapeutic potential across a wide range of conditions, including cardiovascular, neurodegenerative, ophthalmological, renal, metabolic, respiratory, and oncological disorders, among others (63–68). In addition, fasudil is well tolerated by humans, and oral administration is currently being studied in a phase I clinical trial (69). Despite this extensive effort, ROCK inhibitors have not been studied in the context of adrenal disorders, including PA, of which the vast majority of cases are caused by APMs or APAs. Current nonsurgical therapeutic strategies for PA are focused on blocking excess aldosterone signaling or reducing aldosterone production (70). Our study demonstrates that Rho/ROCK inhibition (using fasudil) effectively attenuates zG hyperplasia and reduces aldosterone production by approximately 50%, a therapeutically relevant magnitude that could have meaningful effects on blood pressure and cardiovascular risk in PA. These findings indicate the zG is a direct target for ROCK inhibitors and suggest that ROCK inhibition may offer a promising new therapeutic strategy for treating PA. Finally, the known vasodilatory effects of these drugs may provide additional benefits to hypertensive patients with PA (71).

## Methods

Additional details may be found in Supplemental Methods.

### Sex as a biological variable

Our study examined male and female animals, and similar findings are reported for both sexes. Please see details below and in the main text.

### Animals

Mice were kept on a 12/12-hour light/dark cycle and had free access to standard chow and tap water.

Transgenic βCat-GOF (*Cyp11b2^Cre/+^*:*Ctnnb1^fl(Ex3)/wt^*), αCat-LOF (*Cyp11b2^Cre/+^*:*Ctnna1^fl/fl^*), and βCat-GOF:αCat-LOF (*Cyp11b2^Cre/+^:Ctnnb1^fl(Ex3)/wt^*:*Ctnna1^fl/fl^*) mice were generated by crossbreeding *Cyp11b2^Cre/+^* (33), *Ctnna1^fl/fl^* (72) (Jax mice strain 004604, The Jackson Laboratory) and/or *Ctnnb1^fl(Ex3)/wt^* (73) (a gift from Mark Taketo, Kyoto University, Kyoto, Japan). *Cyp11b2^Cre/+^*:*Ctnnb1^wt/wt^*:*Ctnna1^wt/wt^* mice were used as controls. Littermates were used whenever possible, and efforts were made to avoid genetic drift between control and experimental strains. Mice between 6 and 12 weeks of age were classified as young adult mice, between 16 and 21 weeks of age as adult mice, and between 7 and 12 months of age aged mice. The sex of the mice is specified in the corresponding figure legends.

### In vivo fasudil treatments

#### Young adult mice.

C57BL/6J or littermate controls (*Cyp11b2^Cre/+^*:*Ctnnb1^wt/wt^*) and βCat-GOF mice were injected intraperitoneally with 30 mg/kg fasudil (Selleck Chemicals, dissolved in PBS) every other day for 28 days. Animals injected with PBS served as vehicle controls. The animals were euthanized at the end of the experiments, and adrenals and kidneys were collected for immunofluorescence and mRNA expression analysis. For the last 3 days, C57BL/6J mice were kept individually housed in metabolic cages for 24-hour urinary aldosterone measurements.

#### Adult mice.

Littermate βCat-GOF mice were injected intraperitoneally with 30 mg/kg fasudil or PBS 6 times per week for 28 days. For the last 3 days, mice were kept individually housed in metabolic cages for 24-hour urinary aldosterone measurements. The animals were euthanized at the end of the experiments, and adrenals and kidneys were collected for immunofluorescence and mRNA expression analysis.

#### Aged mice.

Littermate control and βCat-GOF mice were injected intraperitoneally with 30 mg/kg fasudil for 14 days. Three days before treatment and for the last 3 days of treatment, mice were housed individually in metabolic cages for 24-hour urinary aldosterone measurements.

### Urinary aldosterone measurements

Urine for 24-hour periods was collected from mice kept individually housed in metabolic cages and stored at –20°C until assayed. Urinary aldosterone excretion was measured using a competitive RIA aldosterone kit (Tecan) following the manufacturer’s protocol. Briefly, 100 μL of urine was mixed with 1,000 μL of hydrochloric acid and incubated at 30°C for 16 hours. Afterwards, 50 μL of this solution and 150 μL of the Zero Calibrator (Tecan), together with 500 μL of the aldosterone radioactive tracer, were added to aldosterone antibody-coated tubes. After mixing, the tubes were incubated at room temperature for 18 hours. Then, the incubation mixture was decanted, and radioactivity in the tubes was counted using a Cobra II Auto-Gamma counter (Long Island Scientific, Berthold Technologies). Aldosterone levels were normalized to urinary creatinine measured using a calorimetric assay kit (Cayman Chemical).

### Cell culture

The NCI-H295R adrenocortical carcinoma cell line (passage 6–15) was obtained from ATCC and maintained in DMEM-F12 (Gibco) supplemented with 2.5% Nu-Serum (Fisher Scientific), 1× insulin-transferrin-selenium (ITS) (Gibco), 1× Glutamax (Gibco), and 1× penicillin/streptomycin.

### PLA

After deparaffinization and antigen retrieval, adrenal paraffin sections were permeabilized with PBS-T for 5 minutes and blocked with 5% NGS in PBS for 1 hour. The sections were then incubated with KCad and p120 diluted in 5% NGS antibodies overnight at 4°C. PLAs were performed using anti-rabbit plus and anti-mouse minus plus probes combined with the FarRed detection reagents kit (Duolink, Sigma-Aldrich) following the manufacturer’s protocol. Incubations with no primary antibody, only KCad primary antibody, and only p120 primary antibody were used as negative controls.

### Image acquisition and quantification

Imaging was performed using a Zeiss Axio Imager Z2, a Zeiss LSM 510, or a Zeiss LSM 700 confocal laser scanning microscope.

PLA signal intensity in the zG area, determined by the density of nuclei, was calculated after subtracting background using ImageJ software (NIH) and the measure tool.

Equatorial sections of adrenal glands were imaged for analysis when possible, encompassing the capsule, cortical regions, and the medulla. Analysis of the Dab2^+^ area was used as a proxy for the zG and was normalized to the whole cortical area using Zeiss Zen 3.9 software and presented as Dab2^+^ area of cortex (%). The 2D area of glomerular structures, identified by closed circular laminin β1 labeling, was manually quantified using Zeiss Zen 3.9 image analysis software. Rosettes were identified as laminin β1–encapsulated glomerular structures containing ≥5 DAPI-stained nuclei.

The numbers of Ki67^+^ and cleaved caspase-3^+^ cells within the Dab2^+^ area were manually counted and normalized to the total Dab2^+^ area (mm^2^), providing a density of positive cells per unit area.

### Quantification of βCat and KCad signals in human APA samples

APA samples from patients diagnosed with PA were used in this study (Supplemental Table 1). Consecutive paraffin sections of human APA tissue were subjected to immunofluorescence staining for CYP11B2, βCat, and KCad. Whole-slide images of immunostaining for CYP11B2 and βCat were acquired using a Zeiss Axio Imager Z2 microscope. For each sample, random CYP11B2^+^ regions (measuring 255 × 255 μm) were selected in a double-blinded manner to minimize selection bias. Within these regions, the mean βCat and CYP11B2 fluorescence intensity was quantified using the Zen 3.9 analysis tools. In parallel, images of KCad immunostaining were acquired from the same regions using a Zeiss LSM 700 confocal laser scanning microscope under standardized acquisition settings. KCad fluorescence intensity at cell–cell contact points was measured using the line profile tool in ImageJ, with quantification performed in a blinded fashion (as detailed below).

### qLPA

The fluorescence signal intensity was measured along a line (50 pixels wide) drawn between 2 cell nuclei using the line profile tool in ImageJ. Raw data were organized in a tabular format, with each measurement consisting of paired columns representing height and signal values. To facilitate downstream analysis, a custom R function was developed to systematically extract every 2 adjacent columns from the data frame and convert each pair into a separate data frame. This ensured that each pair of height and signal measurements was handled independently for subsequent normalization and analysis.

To enable comparison across datasets with varying height ranges, all height measurements were linearly interpolated to a common scale ranging from 0 to 100 using a custom R function. For each paired data frame, the function identified the minimum and maximum height values and applied a linear transformation to rescale the heights to the standardized interval. Signal values were retained without modification. Data points containing missing values in either the height or signal columns were excluded from further analysis to ensure data integrity.

Following normalization, the list of processed data frames was combined into a single data frame using a custom R function. The resulting data frame contained all normalized height and signal data, structured for efficient batch analysis. To summarize the signal response across the normalized height range, the combined data were partitioned into 1-unit intervals from 0 to 100. The mean signal value within each interval for every measurement pair was computed. For each interval, the function selected all signal values whose corresponding normalized heights fell within the interval and calculated the average, excluding missing values.

Peak signal intensities at each cell–cell interface were averaged per condition per experiment, and the resulting value was used as a single replicate (*n* = number of experiments) for statistical analysis.

### FRAP

The pLV-Puro-EF1A-hCDH6-3xGGGGS-GFP plasmid (KCad-GFP fusion) was obtained from VectorBuilder. Lentiviral particles were generated from this transfer plasmid along with packaging plasmids pLP1, pLP2, and pVSVG in HEK293T cells. NCI-H295R cells were transduced with lentiviral particles in the presence of 5 μg/mL polybrene and incubated for 72 hours. KCad-GFP–expressing cells were then seeded onto poly-l-lysine–coated 35 mm glass-bottom dishes for imaging.

Two days prior to FRAP experiments, media was changed to serum-starved media overnight. Cells were then treated with either NaCl or KCl (15 mM final concentration) for 48 hours in serum-starved media. One hour prior to the experiment, media was changed to imaging buffer containing 5 mM HEPES, 120 mM NaCl, 1.6 mM Na_2_HPO_4_, 5.4 mM NaH_2_PO_4_, 1.3 mM CaCl_2_, 1 mM MgCl_2_, 3 mM KCl, and 15 mM glucose, pH 7.4. For K^+^ stimulated cells, NaCl in the buffer was exchanged with KCl (15 mM final concentration) to maintain osmolarity. Using a Leica TCS SP8 confocal laser scanning microscope with a 63× oil immersion objective, KCad-GFP–expressing cells were monitored using the FRAP wizard. The FRAP protocol consisted of a prebleaching phase for 50 seconds with 5-second intervals (10 frames); a bleaching phase, during which a region of the cell–cell junction was bleached for 1.3 seconds, repeated twice; and a recovery phase for 10 minutes with 5-second intervals (120 frames). FRAP was analyzed by normalizing the recovered intensity in the bleached region of interest relative to the prebleaching and immediate postbleach mean intensities. The normalization formula used was F(*t*)normalized = 100 × [F(*t*) – F0]/[Fpre – F0], where F(*t*) is the fluorescence intensity at time *t*, F0 is the immediate postbleach intensity, and Fpre is the prebleach intensity. Recovery curves were fitted using nonlinear regression in GraphPad Prism 10 to determine the mobile fraction and half-life recovery time (*t_1/2_*).

### Active Rho-GTP pull-down assay

Cells were seeded in 100 mm culture dishes at 80%–90% confluency and serum-starved for 16 hours in DMEM-F12 medium supplemented with 1× ITS. Following starvation, cells were stimulated with KCl (11 mM), AngII (100 nM), or NaCl (11 mM) (vehicle) for 3 hours. Cells were lysed in 250 μL of ice-cold lysis buffer. Using 50 μg of total protein from the cleared lysates, Rho-GTP was pulled down with the RhoA Pull-Down Activation Assay Biochem Kit (Cytoskeleton) following the manufacturer’s protocol.

### Statistics

No power analyses were conducted for this study. All statistical analyses were performed using GraphPad Prism 10 software. No data were excluded from the analysis. Specific details regarding statistical tests and sample sizes (*n*), which represent independent mice, samples, or experiments, are provided in the corresponding figure legends. Data are presented as mean ± SEM unless otherwise indicated. A *P* value less than 0.05 was considered significant.

### Study approval

All animal procedures were approved by Boston Children’s Hospital’s Institutional Animal Care and Use Committee. The studies done with adrenal samples were approved by the ethics commission of the Canton of Zurich, Switzerland (BASEC-Nr. 2017-00771). After obtaining written informed consent, we collected adrenal tissues from patients undergoing adrenalectomy for APAs at the University Hospital Zurich. The diagnosis of PA/APAs was established in accordance with institutional and international guidelines.

### Data availability

Phosphoproteomic data are available in the ProteomeXchange Consortium via the PRIDE partner repository with the dataset identifier PXD027856. Values for all data points in graphs can be found in the Supporting Data Values file. All R scripts used in data analysis and generation of figures are available upon request.

## Author contributions

MB and DTB designed the research. MB, BH, NAG, VC, and KSB conducted experiments. MB, BH, and DTB acquired and analyzed data. FB provided human samples. MB, DLC, and DTB wrote and all authors reviewed and edited the manuscript. MB, FB, PQB, and DTB were responsible for funding acquisition.

## Funding support

This work is the result of NIH funding, in whole or in part, and is subject to the NIH Public Access Policy. Through acceptance of this federal funding, the NIH has been given a right to make the work publicly available in PubMed Central.

- This work was supported by NIH grant 2R01DK123694 (to DTB).
- American Heart Association Fellowship 26POST1569366 (to MB).
- Swiss National Science Foundation (310030L_182700/1) (to FB).
- University Research Priority Program of the University of Zurich (URPP) ITINERARE: Innovative Therapies in Rare Diseases (to FB).

## Supplementary Material

Supplemental data

Unedited blot and gel images

Supporting data values

## Version 1 01/27/2026

In-Press Preview

## Version 2 03/16/2026

Electronic publication
