# Supplementary material for: Rho/ROCK signaling and α-catenin mediate β-catenin–driven hyperplasia in the adrenal cortex via adherens junctions
Source: J Clin Invest. 2026 Jan 27;136(6):e196271. doi: 10.1172/JCI196271 (PMC12987613; doi:10.1172/JCI196271)
Supplement: Supplemental data [file jci-136-196271-s074.pdf]

Supplementary information

Rho-ROCK signaling and α-Catenin mediate β-Catenin-driven hyperplasia in the adrenal via adherens junctions

Mesut Berber[1,2], Betul Haykir[1,2], Nick A. Guagliardo[3], Vasileios Chortis[1,2], Kleiton Silva Borges[1,2], Paula Q. Barrett[3], Felix Beuschlein[4,5,6], Diana L. Carlone[1,2,7], and David T. Breault[1,2,7]

[1]Division of Endocrinology, Boston Children's Hospital, Boston, Massachusetts, USA

[2]Department of Pediatrics, Harvard Medical School, Boston, Massachusetts, USA

[3]Department of Pharmacology, University of Virginia, Charlottesville, VA, 22947, USA

[4]Department of Endocrinology, Diabetology and Clinical Nutrition, University Hospital Zurich (USZ) and University of Zurich (UZH), Zurich, Switzerland

[5]Medizinische Klinik und Poliklinik IV, Klinikum der Universität, Ludwig-Maximilians-Universität, Munich, Germany

[6]The LOOP Zurich - Medical Research Center, Zurich, Switzerland

[7]Harvard Stem Cell Institute, Cambridge, Massachusetts, USA

Contents

Supplementary Figure 1: Rho signaling pathways and adherens junctions are regulated upon secretagogue stimulation. 3

Supplementary Figure 2: Aldosterone secretagogues increase adherens junction stability in NCI-H295R cells. 5

Supplementary Figure 3: β-Catenin Stabilization via CHIR stimulation leads to NCI-H295R cell aggregation and formation of rosette-like structures. 7

Supplementary Figure 4: β-Catenin Stabilization via CHIR stimulation leads to NCI-H295R cell aggregation and formation of rosette-like structures. 9

Supplementary Figure 5: Characterization of adherens junctions in the adrenal cortex of βCat-GOF mice. 11

Supplementary Figure 6: ROCK inhibition with fasudil prevents hyperplasia and reduces aldosterone production. 13

Supplementary Figure 7: Inhibition of βCat's transcription using iCRT14 does not prevent CHIR induced AJ formation. 15

Supplementary Figure 8: zG-specific αCat deletion reduces rosette numbers. 16

Supplementary Figure 9: zG-specific α-Catenin deletion attenuates βCat-GOF-induced zG hyperplasia. 18

Supplementary Figure 10: α-Catenin protects against apoptosis in βCat-GOF adrenals. 19

Supplementary Figure 11: Correlation of β-Catenin and K-Cadherin expression in human aldosterone-producing adenomas. 21

Supplementary Table 1: Information regarding patients harboring aldosterone-producing adenomas (APAs) assessed for β-Catenin and K-Cadherin correlation analysis. 23

Supplementary Table 2: Antibodies list. 24

Supplementary Table 3: TaqMan gene expression assay list. 25

Supplementary Methods 26

- Gene Enrichment Analysis 27
- Cell Dissociation Assays 27
- Cell Proliferation Assay and Nuclear Morphology Assessment 27
- Caspase 3/7 Luminescence Assay 28
- Primary Adrenal Cell Isolation and Culture 28

References 29

Supplementary Figure 1: Rho signaling pathways and adherens junctions are regulated upon secretagogue stimulation.

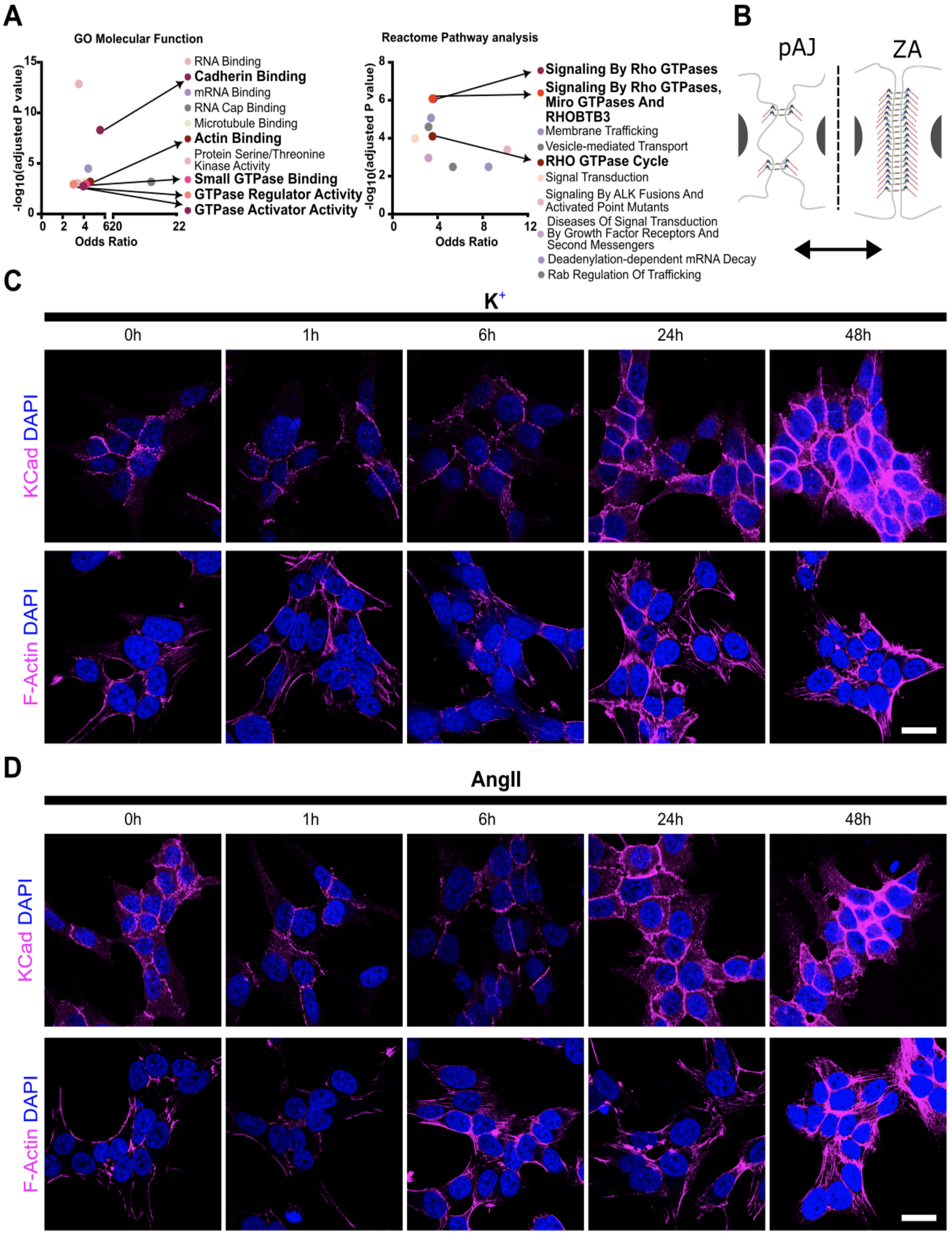

Supplementary Figure 1: Rho signaling pathways and adherens junctions are regulated upon secretagogue stimulation.

A) Significantly enriched molecular function gene ontology (GO) terms and Reactome pathways of dysregulated phosphoproteins upon K^+ stimulation in NCI-H295R cells (1). **B)** Schematic of puncta adherens junctions (pAJ) and zonula adherens (ZA) in epithelial cells. Arrow symbolizes the dynamic continuum between these stuctures. **C,D)** Time-course analysis of K-Cadherin (KCad) and filamentous actin (F-Actin) immunofluorescence upon K^+ (15 mM final concentration) (C) and AngII (100 nM) (D) stimulation (0-48 hr) in NCI-H295R cells. Representative images are shown. Nuclei were counterstained with DAPI and are shown in blue. Scale bar, 20 μm.

Supplementary Figure 2: Aldosterone secretagogues increase adherens junction stability in NCI-H295R cells.

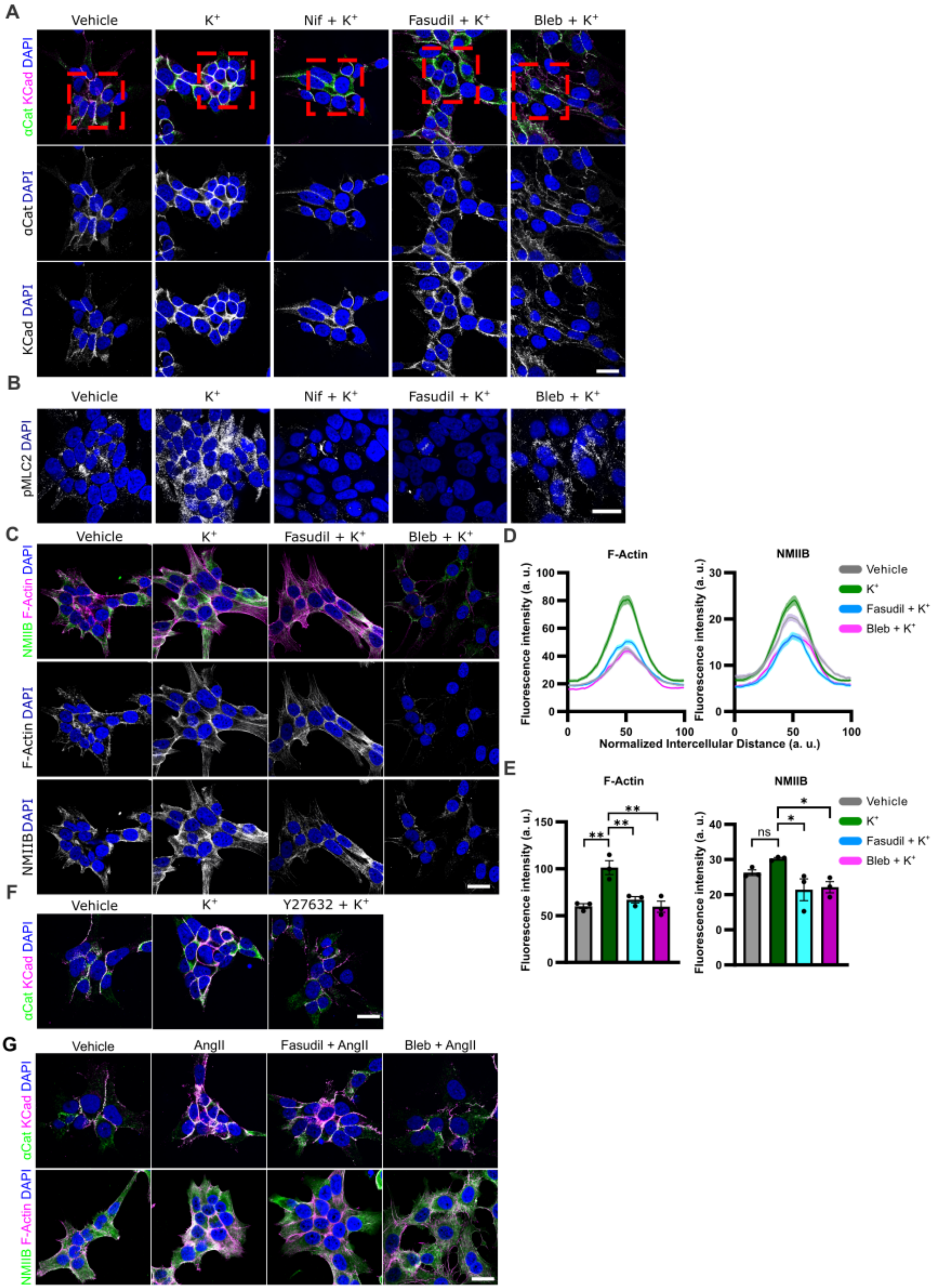

Supplementary Figure 2: Aldosterone secretagogues increase adherens junction stability in NCI-H295R cells.

A) Representative images of α-Catenin (αCat) and K-Cadherin (KCad) immunofluorescence in NCI-H295R cells stimulated with vehicle (11 mM NaCl), K^+ (11 mM KCl) ± nifedipine (Nif, 10 µM) , fasudil (10 µM) or blebbistatin (Bleb, 10 µM) for 48 h (1 h preincubation with inhibitors). Red dashed squares demarcate regions magnified in Figure 1D. **B)** Representative images of phospho-Myosin Light Chain 2 (pMLC2) immunofluorescence in NCI-H295R cells treated as in (A). **C)** Representative images of NMIIB immunofluorescence and F-Actin staining in NCI-H295R cells treated as in (A). **D)** Quantitative line profile analysis of NMIIB and F-Actin fluorescence intensities represented in (C) (n = 90 cell-cell interfaces over three independent experiments). **E)** Average peak fluorescence intensity per experiment for αCat and KCad, corresponding to data in (C) and (D) (n = 3). **F)** Representative images of αCat and KCad immunofluorescence in NCI-H295R cells stimulated with vehicle (11 mM NaCl), K^+ (11 mM) ± Y27632 (50 µM) (1 h preincubation with Y27632). **G)** Representative images of αCat, KCad, NMIIB immunofluorescence and F-Actin staining in NCI-H295R cells stimulated with vehicle (11 mM NaCl), AngII (100 nM) ± Fasudil (10 µM) or blebbistatin (Bleb,10 µM) for 48 h (1 h preincubation with inhibitors). Statistical significance determined by one-way ANOVA with Tukey's multiple-comparison posttest. (*P < 0.05, **P < 0.01, ns , not significant). Nuclei were counterstained with DAPI and are shown in blue. Scale bars, 20 µm.

Supplementary Figure 3: β-Catenin Stabilization via CHIR stimulation leads to NCI-H295R cell aggregation and formation of rosette-like structures.

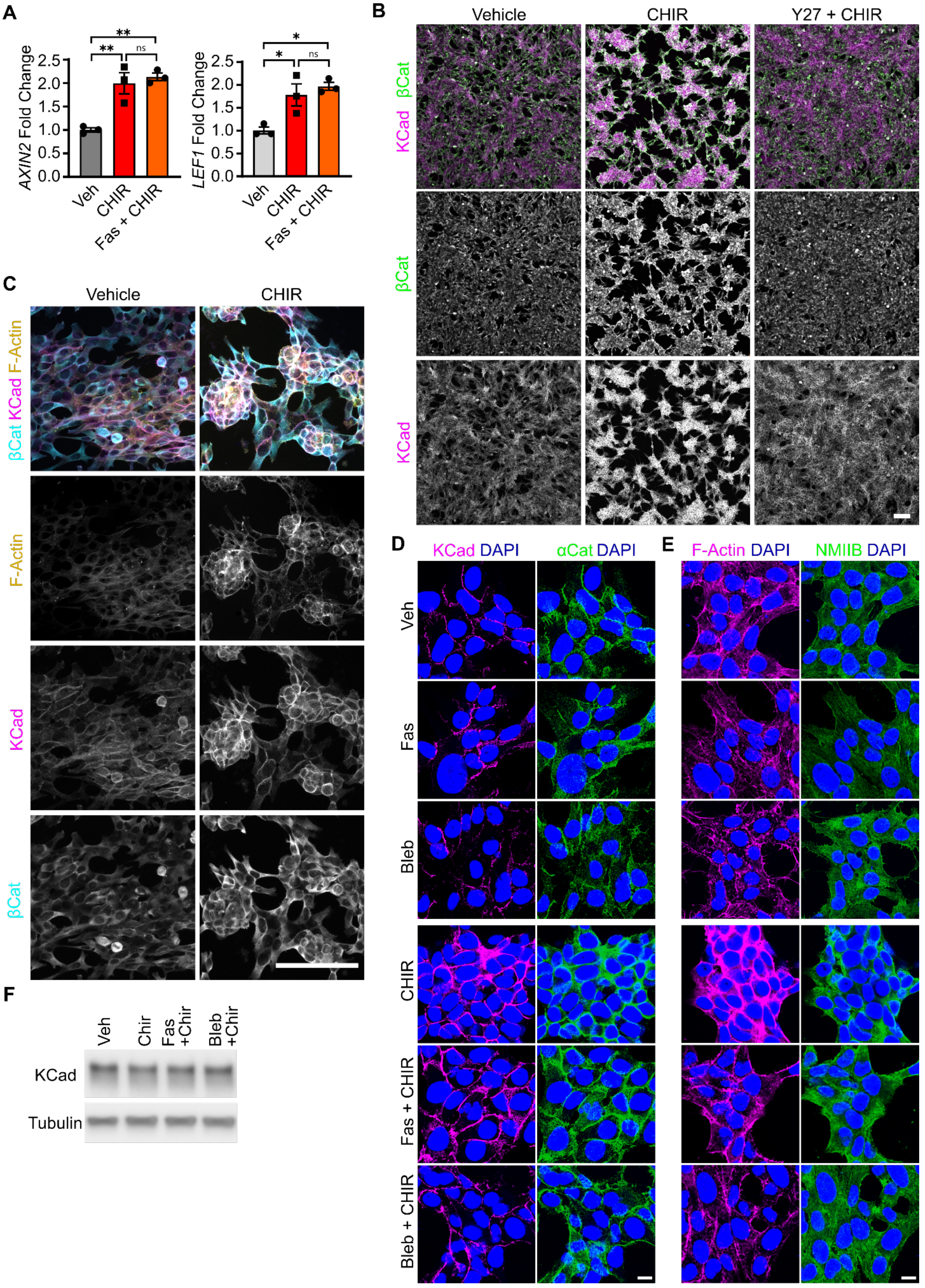

Supplementary Figure 3: ROCK and myosin II activity are required for CHIR-induced adherens junction formation in NCI-H295R cells

A) QPCR analysis of *AXIN2* and *LEF1* mRNA expression in NCI-H295R cells treated with CHIR 99021 (CHIR; 5 μM) ± Fasudil (Fas) (10 μM) or vehicle (DMSO) for 48 h (1 h preincubation with Fas). (n=3) **B)** Representative images of β-Catenin (βCat) and K-Cadherin (KCad) immunofluorescence in NCI-H295R cells stimulated with CHIR (5 μM) ± Y27632 (Y27) (50 μM) or vehicle (DMSO) for 48 h (1 h preincubation with Y27). **C)** Representative Z-stack projections showing βCat and KCad immunofluorescence and F-Actin staining in NCI-H295R cells treated with CHIR (5 μM) or vehicle. **D)** Representative images of α-Catenin (αCat) (green) and K-Cadherin (KCad) (magenta) immunofluorescence with DAPI nuclear counterstain in NCI-H295R cells treated with Vehicle (Veh; DMSO), fasudil (Fas, 10 μM) or blebbistatin (Bleb, 10 μM) and stimulated with Vehicle (Veh; DMSO) or CHIR 99021 (CHIR) (5μM) for 48 h (1 h preincubation with inhibitors), data are displayed as merged images in Figure 4A. **E)** Representative images of Non-muscle myosin IIB (NMIIB) (green) immunofluorescence and filamentous actin (F-Actin) (magenta) staining with DAPI nuclear counterstain in NCI-H295R cells treated as in (D), data are displayed as merged images in Figure 4B. **F)** Immunoblots of K-Cadherin (KCad) and Tubulin in NCI-H295R cells stimulated with vehicle (Veh; DMSO), CHIR (Chir; 5μM) ± fasudil (Fas, 10 μM) or blebbistatin (Bleb; 10 μM) for 48 h (1 h preincubation with inhibitors). Statistical significance determined by one-way ANOVA with Tukey's multiple-comparison posttest. (*P < 0.05, **P < 0.01, ns, not significant). Scale bars, 100 μm or 20 μm (in D and E).

Supplementary Figure 4: β-Catenin Stabilization via CHIR stimulation leads to NCI-H295R cell aggregation and formation of rosette-like structures.

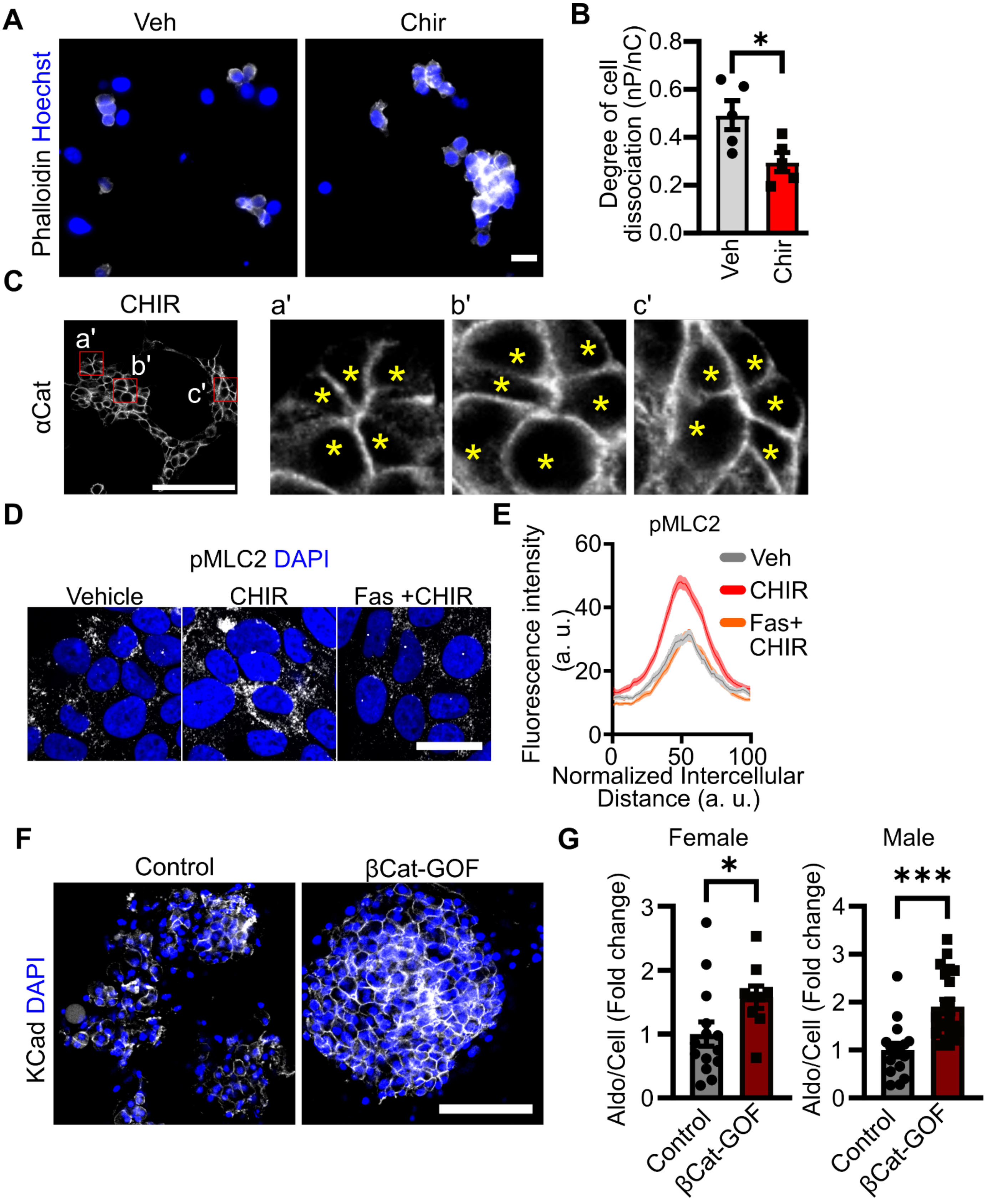

Supplementary Figure 4: β-Catenin Stabilization via CHIR stimulation leads to NCI-H295R cell aggregation and formation of rosette-like structures.

A) Representative images of phalloidin (gray) and Hoechst (blue) staining in NCI-H295R cells stimulated with vehicle (Veh; DMSO) or CHIR (Chir; 5 µM) for 48 h, followed by detachment of confluent cell sheets from culture dishes and application of mechanical stress by 30 cycles of trituration through a Pasteur pipette. **B)** Quantification of the degree of cell dissociation in vehicle- and CHIR-stimulated cells as represented in (E). nP, number of particles; nC, number of cells. (n =5) **C)** Representative confocal images of α-Catenin (αCat) immunofluorescence in NCI-H295R cells stimulated with CHIR. Areas highlighted by red squares are enlarged in panels a', b', and c'. Each asterisk denotes an individual cell within an NCI-H295R rosette-like structure. **D)** Representative confocal images of phospho-Myosin Light Chain 2 (pMLC2) immunofluorescence in NCI-H295R cells with CHIR ± Fasudil (Fas) or vehicle (DMSO) for 48 h (1 h preincubation with Fas). Nuclei were counterstained with DAPI and are shown in blue. **E)** Quantitative line profile analysis of pMLC2 fluorescence intensities represented in (H). Lines represent mean and shaded areas represent standard error of the mean (SEM). (n= Veh, 78; CHIR, 125; Fas + CHIR, 100) **F)** Representative confocal images of KCad immunofluorescence in primary adrenocortical cells from control and β-Catenin gain-of-function (βCat-GOF) mice. Nuclei were counterstained with DAPI and are shown in blue. **G)** Aldosterone production normalized to cell number in primary adrenocortical cells from female and male control and βCat-GOF mice. (n = 14, 9 for female; 16, 21 for male) Statistical significance determined by unpaired two-tailed Student's *t*-test. (*P < 0.05, ***P < 0.001). Data are presented as mean ± SEM. Scale bars, 20 µm (in A and D) or 100 µm (in C and F).

Supplementary Figure 5: Characterization of adherens junctions in the adrenal cortex of βCat-GOF mice.

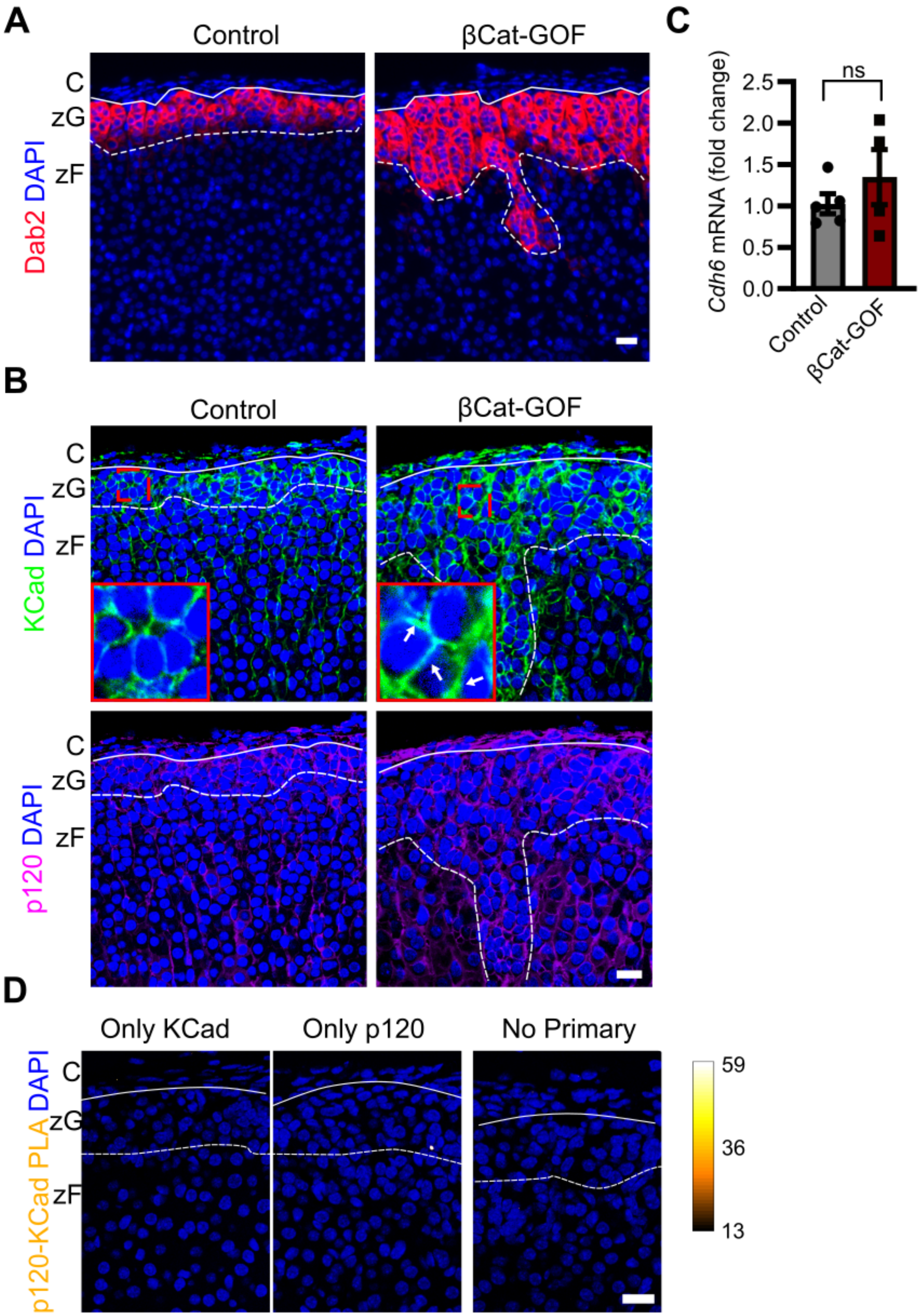

Supplementary Figure 5: Characterization of adherens junctions in the adrenal cortex of βCat-GOF mice.

A) Representative images of Dab2 immunofluorescence in adrenal sections from two-month-old control and βCat-GOF female mice. **B)** Representative images of KCad and p120-Cat (p120) immunofluorescence in adrenal sections from control and βCat-GOF female mice, data are displayed as merged images in Figure 5A. Insets are magnified views of regions marked by dashed red squares. The arrows denote the zonula adherens. **C)** K-Cadherin (*Cdh6*) mRNA expression in adrenals of six-week-old control and βCat-GOF male mice assessed by quantitative PCR (n = 5, 4 mice). Statistical significance determined by unpaired two-tailed Student's *t*-test (ns, not significant). **D)** Representative images of proximity ligation assay negative controls for Figure 4B. The fluorescence signal is represented as a heat map using an orange-hot lookup table. Solid lines mark the boundary between the adrenal capsule (C) and zona Glomerulosa (zG), while dashed lines indicate the boundary between the zG and zona fasciculata (zF). Nuclei were counterstained with DAPI and are shown in blue. Scale bars, 20 μM.

Supplementary Figure 6: ROCK inhibition with fasudil prevents hyperplasia and reduces aldosterone production.

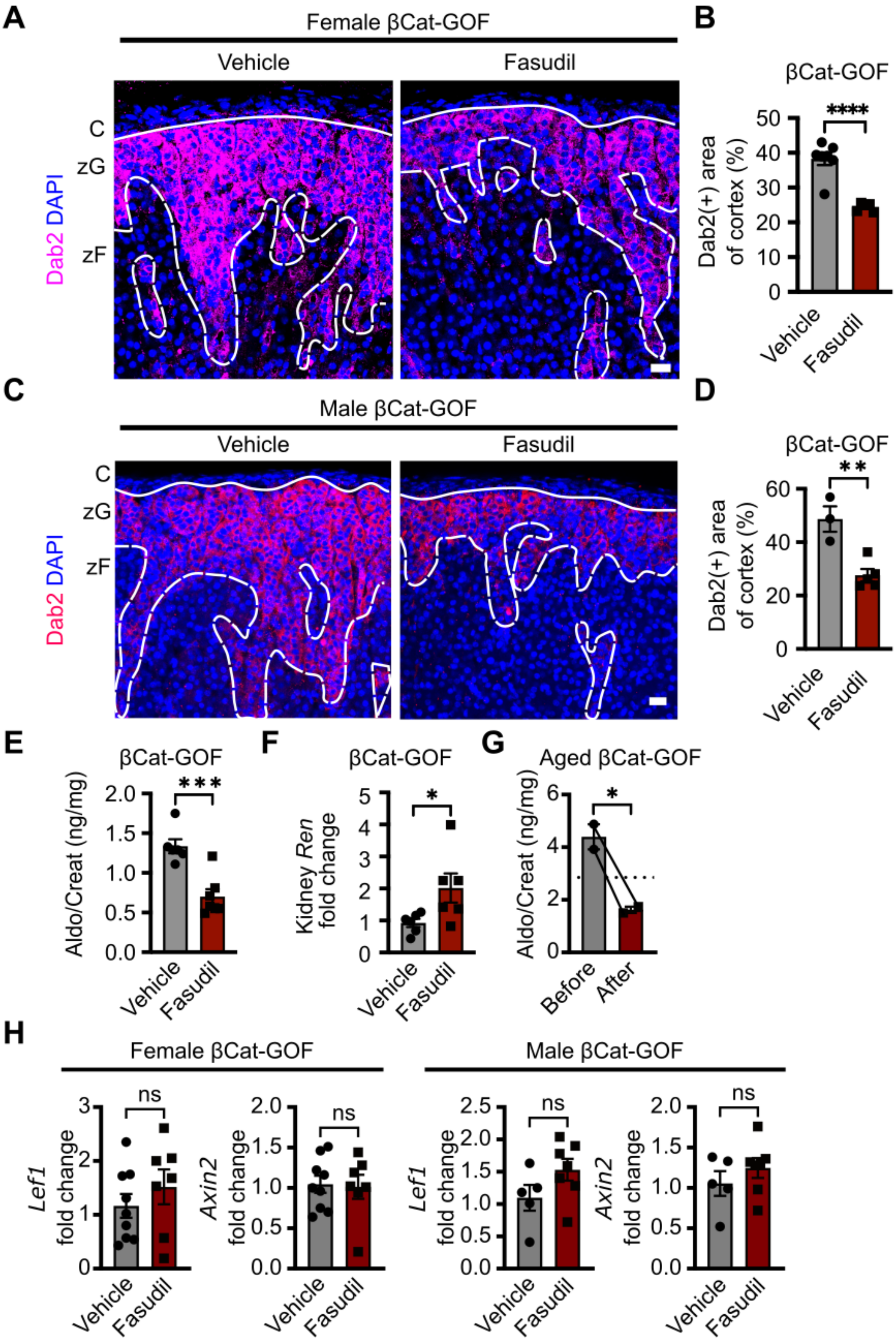

Supplementary Figure 6: ROCK inhibition with fasudil prevents hyperplasia and reduces aldosterone production.

A) Representative images of Dab2 immunofluorescence in adrenal sections from four-month-old βCat-GOF female mice treated with either Vehicle (PBS) or Fasudil (30 mg/kg) administered six times per week for 28 days. **B)** Quantification of the percentage of Dab2(+) area in the adrenal cortex, as shown in (A) (n = 7, 5 mice). **C)** Representative images of Dab2 immunofluorescence in adrenal sections from four-month-old βCat-GOF male mice treated as in (A). **D)** Quantification of the percentage of Dab2(+) area in the adrenal cortex, as shown in (C) (n = 3, 5 mice). **E)** Measurement of 24-hour urinary aldosterone (Aldo) levels in four-month-old βCat-GOF male mice treated as in (A), assessed by radioimmunoassay (RIA) and normalized to creatinine (Creat) (n = 6, 7 mice). **F)** *Renin* (*Ren*) mRNA expression in kidneys of four-month-old βCat-GOF male mice treated as in (A), assessed by qPCR (n = 6, 6 mice). **G)** Measurement of 24-hour urinary aldosterone (Aldo) levels in one-year-old Aged βCat-GOF male mice before and after fasudil treatment (30 mg/kg) administered 6 times weekly for 14 days, assessed by RIA and normalized to creatinine (Creat) (n = 2 mice). Dotted line indicates mean aldosterone levels in untreated littermate controls. Data points for controls are presented in Fig. 2H. **H)** *Lef1* and *Axin2* mRNA expression in adrenals of vehicle- and fasudil-treated four-month-old βCat-GOF female and male mice, assessed by qPCR (n = 5, 9 mice). Statistical significance determined by unpaired two-tailed Student's *t*-test or ratio paired t-test (in G) (*P < 0.05, **P < 0.01, ***P < 0.001, ****P < 0.0001, ns, not significant.) Data are presented as mean ± SEM. Solid white lines mark the boundary between the adrenal capsule (C) and zona glomerulosa (zG), while dashed white lines indicate the boundary between the zG and zona fasciculata (zF). Nuclei were counterstained with DAPI and are shown in blue. Scale bars, 20 µm.

Supplementary Figure 7: Inhibition of βCat's transcription using iCRT14 does not prevent CHIR induced AJ formation.

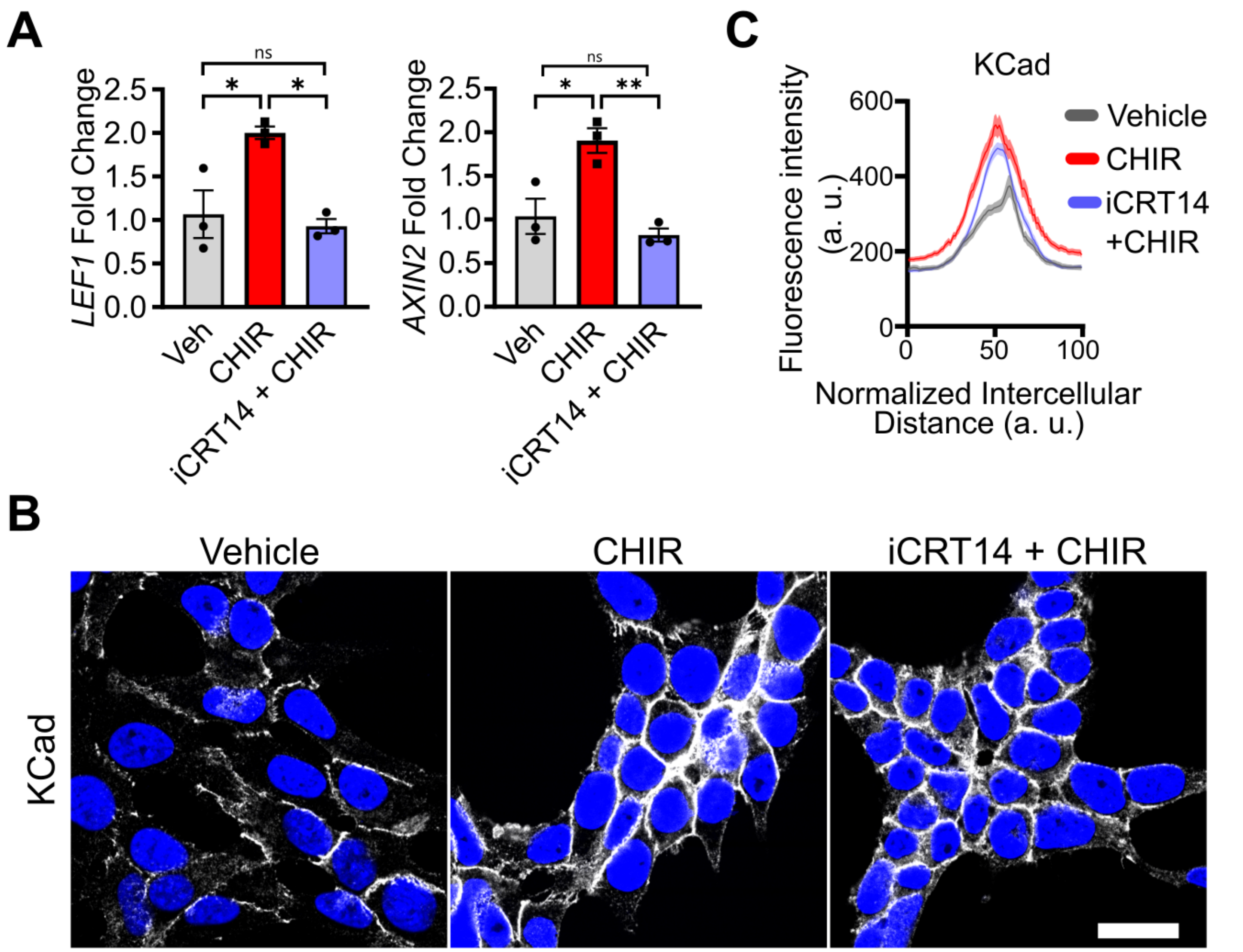

Supplementary Figure 7: Inhibition of βCat's transcription using iCRT14 does not prevent CHIR induced AJ formation.

A) QPCR analysis of *LEF1* and *AXIN2* mRNA expression in NCI-H295R cells treated with CHIR 99021 (CHIR; 5 µM) ± iCRT14 (20 µM) or vehicle (DMSO) for 48 h (1 h preincubation with iCRT14). **B)** Representative images of K-Cadherin (KCad) immunofluorescence in NCI-H295R treated as in A. **C)** Quantitative line profile analysis of KCad fluorescence intensities represented in (B). (n=84, 111, 146 cell-cell interfaces). Lines represent mean and shaded areas represent standard error of the mean (SEM). Statistical significance determined by one-way ANOVA with Tukey's multiple-comparison posttest. (*P < 0.05, **P < 0.01, ns, not significant). Nuclei were counterstained with DAPI and are shown in blue. Scale bars, 20 µM.

Supplementary Figure 8: zG-specific αCat deletion reduces rosette numbers.

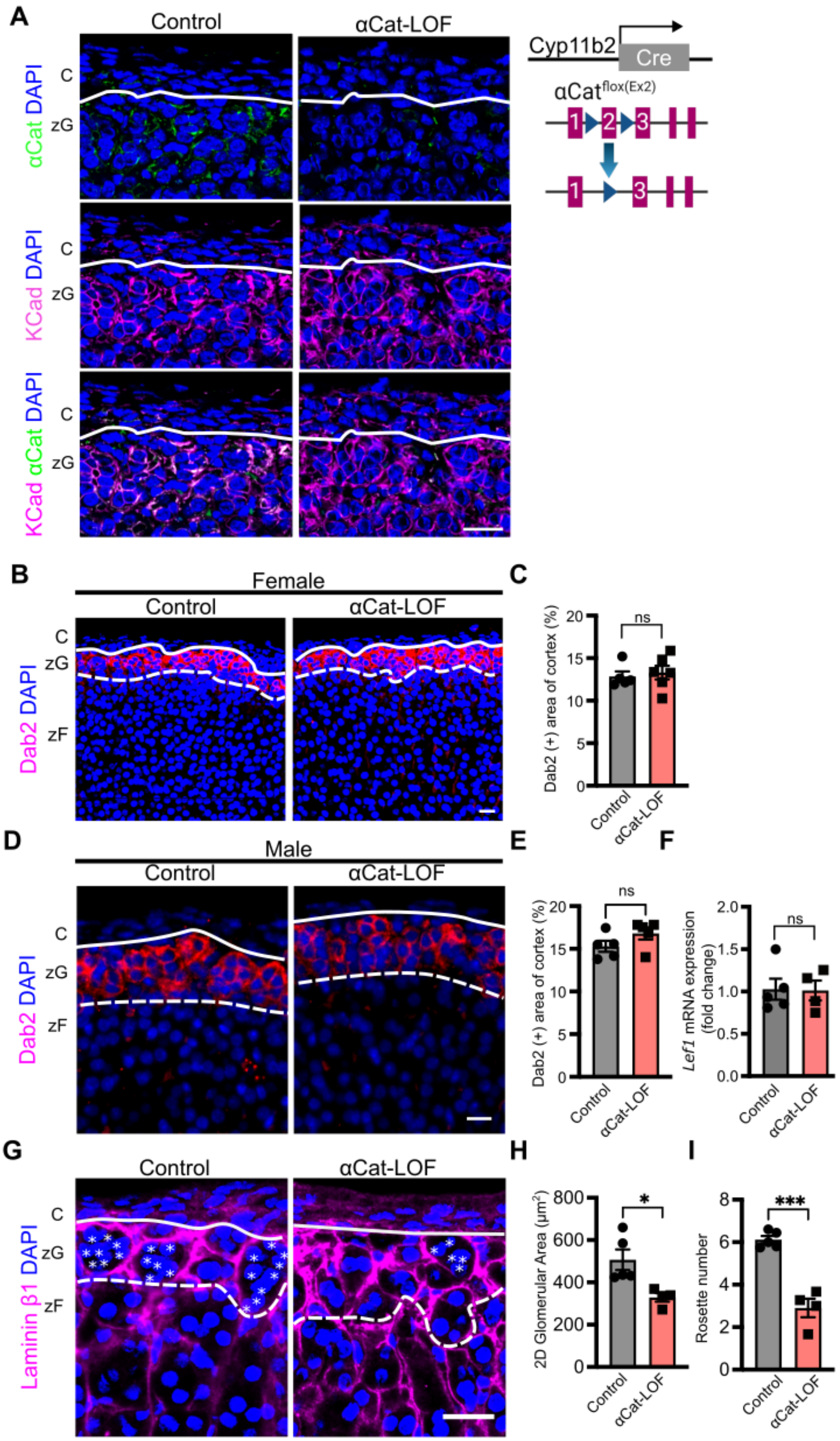

Supplementary Figure 8: zG-specific αCat deletion reduces rosette numbers.

C) Representative images of αCat and KCad immunofluorescence of adrenal sections from two-month-old control and αCat-LOF male mice. Schematic of Cyp11b2$^{Cre/+}$::Ctnna1$^{flox/flox}$ (αCat-LOF) mice is shown to the right. **D)** Representative images of Dab2 immunofluorescence in adrenal sections from two-month-old control and αCat-LOF female mice. **E)** Quantification of Dab2(+) percentage of the adrenal cortex represented in (D) (n = 5, 6 mice). **F)** Representative images of Dab2 immunofluorescence in adrenal sections from two-month-old control and αCat-LOF male mice. **G)** Quantification of Dab2(+) percentage of the adrenal cortex represented in (F) (n = 5, 5 mice). **H)** *Lef1* mRNA expression in adrenals of two-month-old control and αCat-LOF male mice assessed by quantitative PCR (n = 4, 5 mice). **(I)** Representative images of Laminin β1 immunofluorescence in adrenal sections from two-month-old control and αCat-LOF male mice. Each white asterisk denotes an individual cell within a zG rosette. **J)** Quantification of the two-dimensional (2D) area of glomerular structures, as defined by Laminin β1 labeling in (I) (n = 5, 4 mice). **K)** Quantification of rosette number, defined as clusters of five or more cells within a single glomerular structure in regions measuring 200 x 200 μm, as shown in (I). Each asterisk denotes an individual cell within a zG rosette (n = 5, 4 mice). All statistical significance determined by unpaired two-tailed Student's *t*-test (*P < 0.05, ***P < 0.001, ns, not significant). Data are presented as mean ± SEM. Solid white lines mark the boundary between the adrenal capsule (C) and zona glomerulosa (zG), while dashed white lines indicate the boundary between the zG and zona fasciculata (zF). Nuclei were counterstained with DAPI and are shown in blue. Scale bars, 20 μM.

Supplementary Figure 9: zG-specific α-Catenin deletion attenuates βCat-GOF-induced zG hyperplasia.

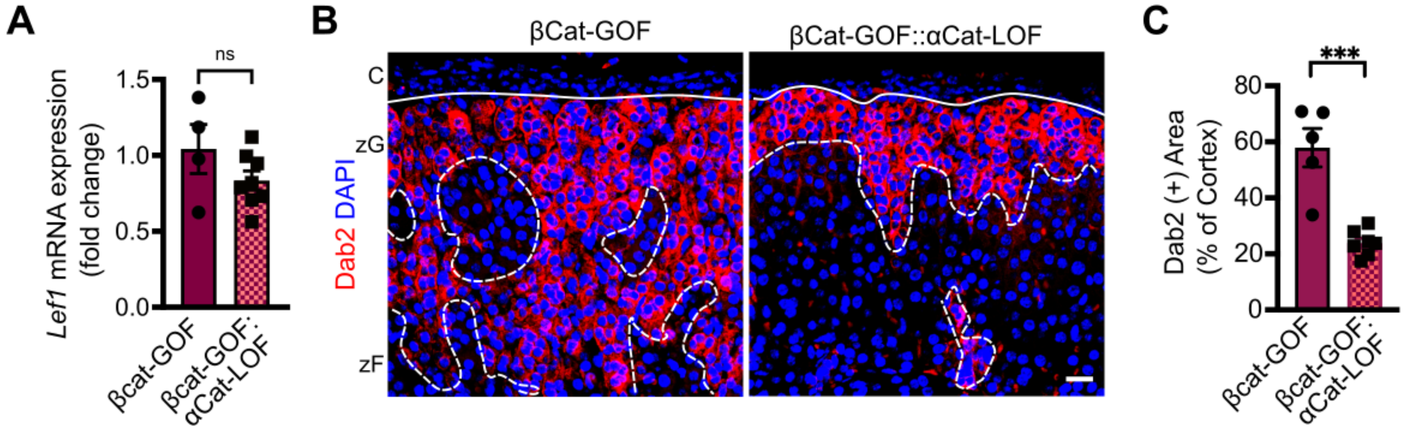

Supplementary Figure 9: zG-specific α-Catenin deletion attenuates βCat-GOF-induced zG hyperplasia.

A) *Lef1* mRNA expression in adrenals of two-month-old βCat-GOF and βCat-GOF::αCat-LOF male mice assessed by quantitative PCR (n = 4, 8 mice). **B)** Representative images of Dab2 immunofluorescence in adrenal sections from four-month-old βCat-GOF and βCat-GOF::αCat-LOF female mice. **C)** Quantification of the percentage of Dab2(+) area in the adrenal cortex, represented in (B) (n = 5, 7 mice). Statistical significance determined by unpaired two-tailed Student's *t*-test (***P < 0.001, ns, not significant). Data are presented as mean ± SEM. Solid white lines mark the boundary between the adrenal capsule (C) and zona glomerulosa (zG), while dashed white lines indicate the boundary between the zG and zona fasciculata (zF). Nuclei were counterstained with DAPI and are shown in blue. Scale bar, 20 μM.

Supplementary Figure 10: α-Catenin protects against apoptosis in βCat-GOF adrenals.

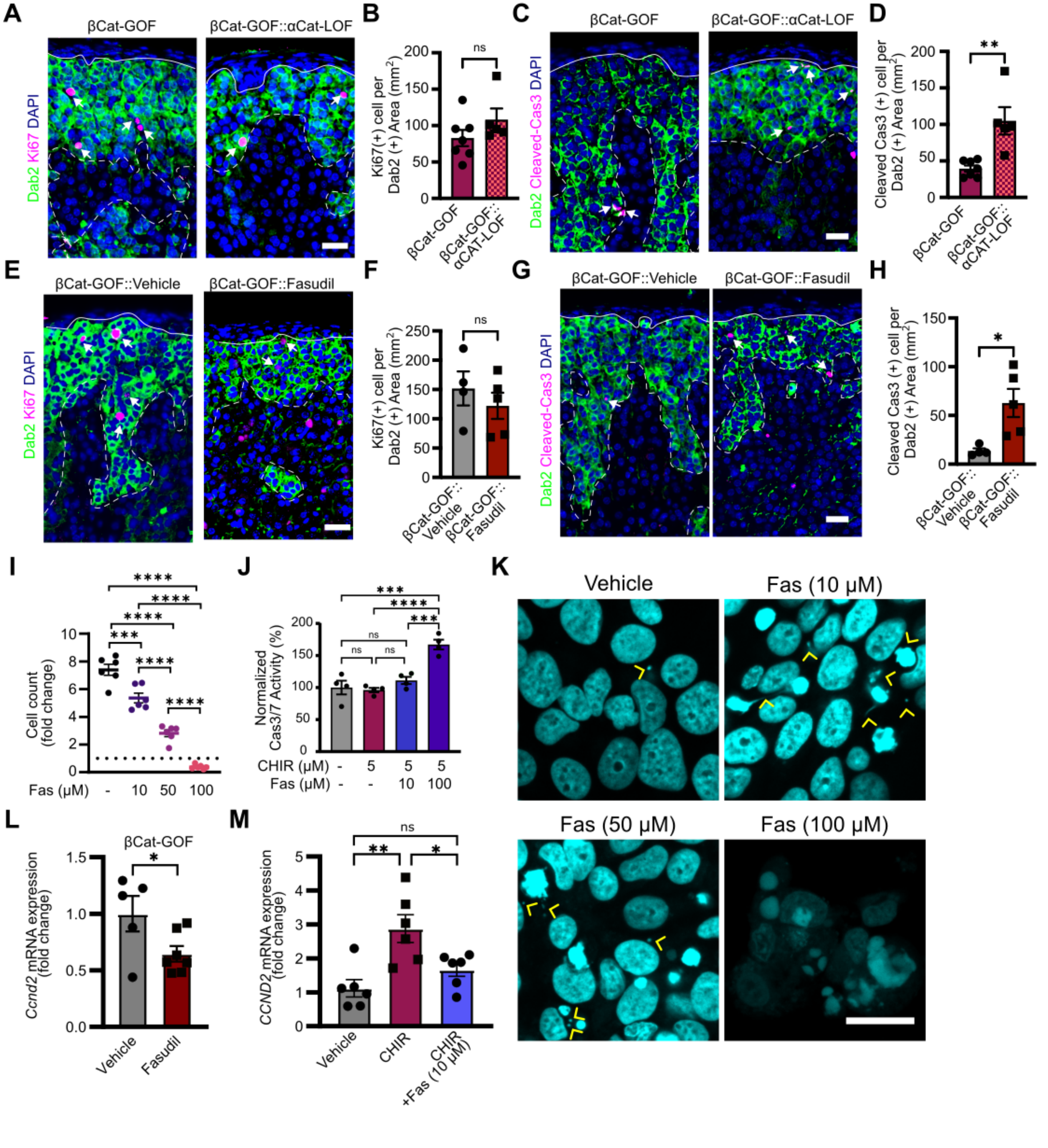

Supplementary Figure 10: α-Catenin protects against apoptosis in βCat-GOF adrenals.

A) Representative images of Dab2 and Ki67 immunofluorescence in adrenal sections from four-month-old βCat-GOF and βCat-GOF::αCat-LOF male mice. **B)** Quantification of Ki67(+) cells in the Dab2(+) area, represented in (A) (n = 7, 5 mice). **C)** Representative images of Dab2 and cleaved-Caspase 3 immunofluorescence in adrenal sections from four-month-old βCat-GOF and βCat-GOF::αCat-LOF male mice. **D)** Quantification of cleaved-Caspase 3-positive cells in Dab2(+) area, represented in (C) (n = 7, 5 mice). **E)** Representative images of Dab2 and Ki67 immunofluorescence in adrenal sections from four-month-old βCat-GOF male mice treated with either Vehicle (PBS) or Fasudil (30 mg/kg) administered six times per week for 28 days. **F)** Quantification of Ki67(+) cells in the Dab2(+) area, represented in (E) (n = 4, 5 mice). **G)** Representative images of Dab2 and cleaved-Caspase 3 immunofluorescence in adrenal sections from four-month-old βCat-GOF male mice treated as in (E). **H)** Quantification of cleaved-Caspase 3(+) cells in the Dab2(+) area, represented in (G) (n = 4, 5 mice) **I)** Cell counts of NCI-H295R cells cultured for 10 days with vehicle or fasudil (Fas; 10, 50, and 100 μM). Data are normalized to seeding number and represented as fold change. The dotted line indicates the normalized seeding count. **J)** Caspase 3/7 luminescence activity measured in NCI-H295R cells treated with CHIR (5 μM) ± fasudil (Fas; 10 and 100 μM) or vehicle (DMSO) for 6 h (1 h preincubation with fasudil), using the Caspase-Glo 3/7 Assay System. **K)** Representative images of NCI-H295R cell nuclei treated as in (I). Yellow arrowheads indicate abnormally segregated nuclei. **L)** Cyclin D2 (*Ccnd2*) mRNA expression in adrenals of vehicle- and fasudil-treated four-month-old βCat-GOF male mice, assessed by quantitative PCR (n = 5, 7 mice). **M)** *CCND2* mRNA expression in NCI-H295R cells treated with CHIR (5 μM) ± fasudil (Fas; 10 μM) or vehicle (DMSO) for 48 h (1 h preincubation with fasudil). Statistical significance determined by unpaired two-tailed Student's *t*-test or one-way ANOVA with Tukey's multiple-comparison posttest (in I) (*$P < 0.05$, **$P < 0.01$, ***$P < 0.001$, ****$P < 0.0001$, ns, not significant). Solid white lines mark the boundary between the adrenal capsule (C) and zona glomerulosa (zG), while dashed white lines indicate the boundary between the zG and zona fasciculata (zF). Nuclei were counterstained with DAPI and are shown in blue. Scale bars, 20 μM.

Supplementary Figure 11: Correlation of β-Catenin and K-Cadherin expression in human aldosterone-producing adenomas.

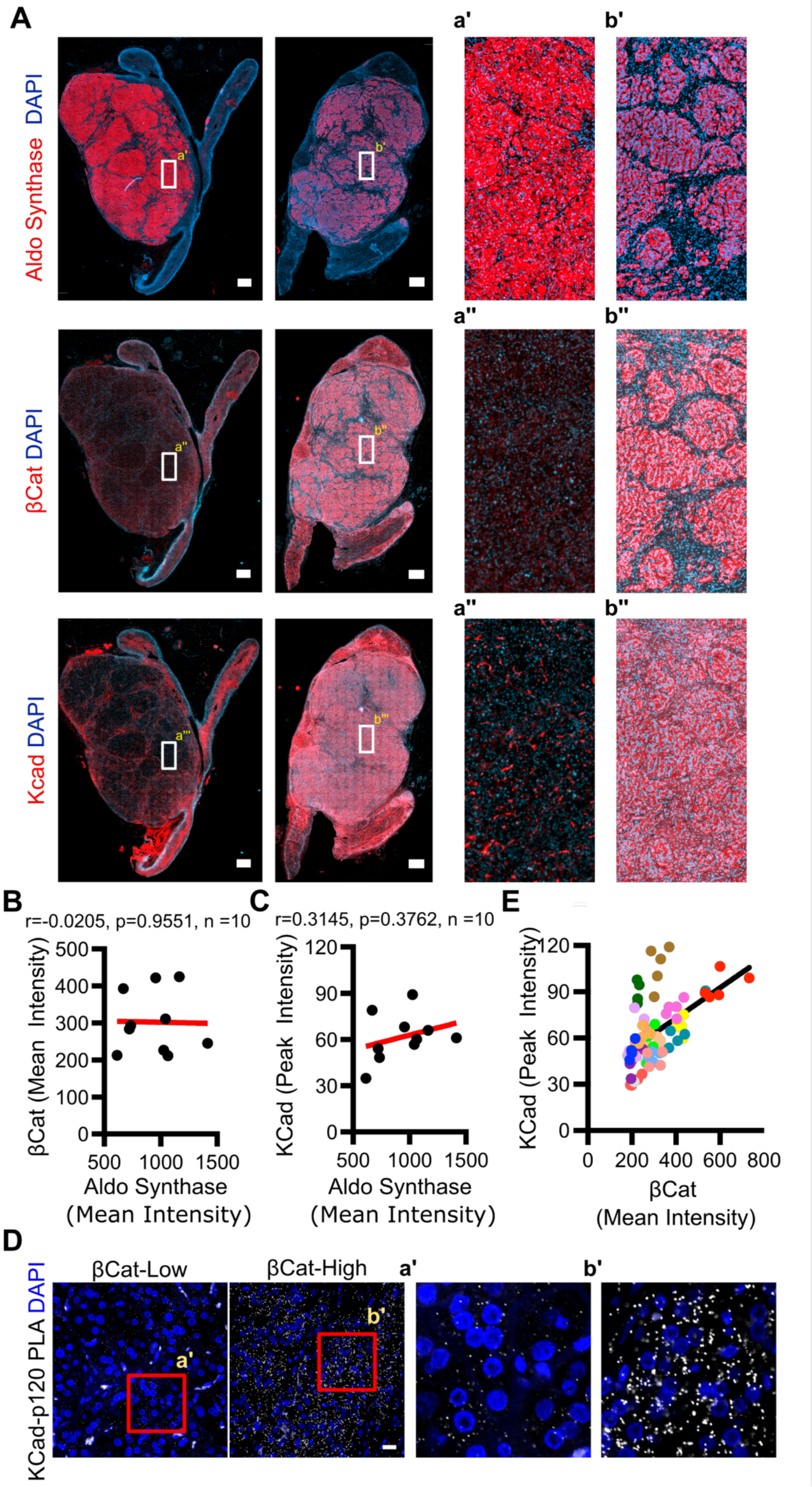

Supplementary Figure 11: Correlation of β-Catenin and K-Cadherin expression in human aldosterone-producing adenomas.

A) Representative low-magnification immunofluorescence images showing aldosterone (Aldo) synthase, β-Catenin (βCat) and K-Cadherin (KCad) labeling in human aldosterone-producing adenoma specimens from Fig. 6A,B. Areas highlighted by white rectangles are enlarged in panels a', b', a”, b''. Scale bars, 1 mm. **B,C)** Pearson correlation analysis comparing βCat mean intensity and Aldo synthase mean intensity **(B)** and K-Cadherin (KCad) mean intensity and Aldo synthase mean intensity **(C)** from 10 human APA samples. The red lines represent the regression fit. **D)** Representative images of KCad/p120 proximity ligation assay (PLA) signal in human aldosterone-producing adenoma sections. Areas highlighted by red rectangles are shown at higher magnification in panels a' and b'. Scale bar, 20 μM. **E)** Scatter plot showing quantification from each randomly selected region (n=3-5) for every tumor section, with different colors representing different individuals, from the analysis in Fig. 6D comparing βCat mean intensity and KCad peak intensity. Nuclei were counterstained with DAPI and are shown in blue.

Supplementary Table 1: Information regarding patients harboring aldosterone-producing adenomas (APAs) assessed for β-Catenin and K-Cadherin correlation analysis.

Patient #	Sex	Age	Diagnosis
1	Female	30	Primary Aldosteronism (APA)
2	Female	38	Primary Aldosteronism (APA)
3	Male	40	Primary Aldosteronism (APA)
4	Male	42	Primary Aldosteronism (APA)
5	Male	49	Primary Aldosteronism (APA)
6	Female	49	Primary Aldosteronism (APA)
7	Male	50	Primary Aldosteronism (APA)
8	Male	51	Primary Aldosteronism (APA)
9	Male	51	Primary Aldosteronism (APA)
10	Male	52	Primary Aldosteronism (APA)
11	Female	54	Primary Aldosteronism (APA)
12	Male	56	Primary Aldosteronism (APA)
13	Male	57	Primary Aldosteronism (APA)
14	Female	61	Primary Aldosteronism (APA)
15	Male	64	Primary Aldosteronism (APA)
16	Male	68	Primary Aldosteronism (APA)

Supplementary Table 2: Antibodies list.

Antibody	Host	Source	Catalog #	Used for	Dilutions
anti-α-Catenin	Mouse	Invitrogen	13-9700	IP, IF, WB	1:50, 1:100, 1:1000
anti-K-Cadherin	Rabbit	Abcam	ab133632	IF, WB, PLA	1:200, 1:1000, 1:200
Anti-pMLC2 (Ser 19)	Rabbit	Cell Signaling Technologies	3671S	IF	1:50
anti-non-muscle-myosin II-B	Rabbit	BioLegend	909901	IF	1:200
anti-Laminin β1	Rat	Santa Cruz	sc-33709	IF	1:100
anti-β-Catenin	Mouse	BD Biosciences	610154	IF	1:200
anti-p120-Catenin	Mouse	BD Biosciences (Fisher Scientific)	BDB610133	IF, PLA	1:100, 1:100
anti-p120-Catenin	Mouse	Assay Biotech	YM4837	PLA (Human)	1:100
anti-Dab2	Rabbit	Cell Signaling Technologies	12906	IF	1:200
anti-Dab2	Mouse	BD Biosciences	610464	IF	1:100
anti-Ki67	Rabbit	Cell Signaling Technologies	12202	IF	1:100
anti-cleaved-Caspase3	Rabbit	Cell Signaling Technologies	9661	IF	1:100
anti-Cyp11b2	Mouse	Dr. Celso E. Gomez-Sanchez, University of Mississippi Medical Center, Jackson, MS, USA	hCYP11B2-41-13B	IF	1:1000
anti-β-Actin	Mouse	Santa Cruz	sc-47778	WB	1:5000
anti-α-Tubulin	Mouse	Santa Cruz	sc-32293	WB	1:1000
anti-Rabbit IgG-647	Goat	Invitrogen	A21245	IF	1:400
anti-Rabbit IgG-488	Goat	Invitrogen	A11008	IF	1:400
anti-Mouse IgG-488	Donkey	Invitrogen	A21202	IF	1:400
anti-Mouse IgG-647	Donkey	Invitrogen	A31571	IF	1:400
anti-Rat IgG-647	Goat	Invitrogen	A21247	IF	1:400
anti-Rabbit IgG-HRP	Goat	Cell Signaling Technologies	7074	WB	1:2000
anti-Mouse IgG-HRP	Horse	Cell Signaling Technologies	7076	WB	1:5000

Supplementary Table 3: TaqMan gene expression assay list.

Species	Gene	Assay catalog number
Human	*18S*	Hs99999901_s1
Human	*AXIN2*	Hs00610344_m1
Human	*LEF1*	Hs01547250_m1
Huamn	*CCND2*	Hs00153380_m1
Mouse	*Gapdh*	Mm99999915_g1
Mouse	*18s*	Mm02601777_g1
Mouse	*Axin2*	Mm00443610_m1
Mouse	*Lef1*	Mm00550265_m1
Mouse	*Cdh6*	Mm01310024_m1
Mouse	*Ccnd2*	Mm00438070_m1
Mouse	*Renin*	Mm02342887_mH
Mouse	*Cyp11b2*	Mm01204955_g1

Supplementary Methods

Immunofluorescence assays

Antibodies used in these studies are listed in **Supplementary Table 2.**

Cell culture: NCI-H295R cells were seeded onto poly-L-lysine (Sigma)-coated coverslips placed in 24-well plates at a density of 2 × 10^5 Following one day in culture, cells were serum-starved in DMEM-F12 medium containing 1X ITS, 1X Glutamate, and 1X penicillin/streptomycin for 16 hours. Cells were then treated for 1 hour with either 10 µM (–) blebbistatin (StemCell Technologies), 50 µM Y27632 (Tocris), 10 µM fasudil (Selleckchem), 10 µM nifedipine (Sigma), 20 µM iCRT14 (MedChemExpress) or DMSO as a control. Following pretreatment, cells were stimulated with either 11 mM KCl (15 mM final concentration), 100 nM AngII, 5 µM CHIR 99021, or vehicle controls (11 mM NaCl for KCl and DMSO for the small molecules). AngII was diluted in an 11 mM NaCl solution, allowing NaCl to serve as the vehicle control for both KCl and AngII treatments.

After 48 hours of stimulation (unless otherwise specified), cells were fixed with 4% paraformaldehyde (PFA) for 15 minutes at room temperature and washed three times with PBS. Cells were permeabilized with PBS-0.1%Tween (PBS-T) for 15 minutes and blocked with 1% bovine serum albumin (BSA) in PBS-T for 20 minutes. Samples were incubated with primary antibodies diluted in blocking buffer for 4 hours at room temperature. After three PBS washes, cells were incubated for 2 hours with fluorophore-conjugated secondary antibodies , far-red fluorescent phalloidin conjugate (1:1000; Life Technologies), CellMask Actin Tracking stain (Invitrogen), and 4′,6-diamidino-2-phenylindole (DAPI; 1:1000; Sigma). Finally, coverslips were washed three times with PBS and mounted onto glass slides using ProLong Gold antifade mountant (Invitrogen).

Thick sections: For imaging of rosette structures, adrenal glands were collected from mice and immediately placed in cold PBS. After carefully removing the surrounding fat tissue, the glands were cut in half with a blade and fixed overnight at 4°C in 4% PFA. The fixed adrenals were then embedded in 4% low-melting temperature agarose, and 70-µm-thick sections were prepared using a Leica Vibratome and transferred to 48-well plates. Adrenal sections were permeabilized with PBS-T for 30 minutes and then blocked with a solution of 1% BSA and 5% normal goat serum (NGS) (Sigma) in PBS-T for one hour at room temperature (RT). Samples were incubated with primary antibodies diluted in blocking buffer overnight at 4°C. After three washes with PBS-T, sections were incubated for two hours with fluorophore-conjugated secondary antibodies and DAPI. Following three additional washes with PBS-T, adrenal slices were mounted on slides using ProLong Gold antifade mountant (Invitrogen).

Paraffin sections: Immunofluorescent analysis was performed using established protocols, as described previously (2). Briefly, after fixation, adrenals were dehydrated in ethanol and embedded in paraffin blocks. Paraffin sections were cut at 5 µm thickness. Sections were rinsed in xylene, an ethanol gradient and then PBS. Antigen retrieval was performed in Tris-EDTA pH 9.0. Sections were blocked in 5% NGS, 0.1% Tween-20 in PBS for 1 h at RT. Primary antibodies were diluted in 5% NGS in PBS and incubated on sections at 4 °C overnight. Slides were washed three times for 5 min in 0.1% Tween-20 in PBS. Secondary antibodies were diluted in 1% BSA in PBS and incubated on sections at RT for 1–2 h. For nuclear staining, DAPI (4′,6-diamidino-2-phenylindole) was added to the secondary antibody mixture at a final concentration of 1:1000. After three 5 min washes with 0.1% Tween-20 in PBS, slides were mounted with ProLong Gold antifade mountant.

RNA isolation and qPCR

NCI-H295R cells were plated in 12-well plates at a density of 5 × 10^5. Following one day in culture, cells were then pretreated for one hour with 20 µM iCRT14, 10 µM fasudil, or DMSO as the control. Following pretreatment, cells were stimulated with either 5 µM CHIR 99021 or vehicle (DMSO) for 48 hours. Whole tissue (adrenal, kidney) was homogenized with a tissue homogenizer, and total RNA and cDNA were prepared as described previously (1). Gene expression analysis was assessed by TaqMan Universal PCR Master Mix (Applied Biosystems) and QuantStudio 6 Flex thermocycler (Applied Biosystems). Taqman probes are listed in **Supplementary Table 3.**

Western blot analyses

Protein lysates prepared in Laemmli buffer were boiled at 95°C for 10 minutes, separated by sodium dodecyl sulfate-polyacrylamide gel electrophoresis (SDS-PAGE), and transferred to polyvinylidene difluoride (PVDF) membranes. Membranes were blocked with either 1% BSA or 5% skim milk powder in PBS-T for 15 minutes, then incubated with primary antibodies overnight at 4°C. After three washes with PBS-T, membranes were incubated with horseradish peroxidase (HRP)-conjugated secondary antibodies in blocking solution for 1 hour at room temperature. Following three additional PBS-T washes, membranes were developed using enhanced chemiluminescence (ECL) substrate. Chemiluminescent signals were detected either by exposure to X-ray film or by imaging with a ChemiDoc Imaging System (Bio-Rad). Densitometric analysis of band intensities was performed using ImageJ software.

For analysis of NCI-H295R cells, cells were seeded in 6 well plates at a density of 1×10^6. Following one day in culture, cells were serum-starved in DMEM-F12 medium containing 1X ITS, 1X Glutamate, and 1X penicillin/streptomycin for 16 hours. Cells were then treated for 1 hour with either 10 µM (–) blebbistatin (StemCell Technologies), 10 µM fasudil (Selleckchem), or DMSO as a control. Following pretreatment, cells were stimulated with 5 µM CHIR 99021, or vehicle (DMSO) for 48 hours. Cells were collected by scraping from 6-well plates with cell lysis buffer (200 mM EPPS pH 8.5, 8M Urea, 0.5% SDS, Protease/Phosphatase Inhibitor Cocktail (Cell Signaling Technologies)).

Coimmunoprecipitation

Cells were seeded in 100 mm culture dishes at 80-90% confluency and serum-starved for 16 hours in DMEM/F12 medium supplemented with 1× ITS. Following starvation, cells were stimulated with KCl (11 mM), AngII (100 nM) or NaCl (11 mM) (vehicle) for 48 hours. After stimulation, cells were lysed on ice using 500 µl IP lysis buffer supplemented with protease and phosphatase inhibitors (Santa Cruz Biotechnology). Lysates were clarified by centrifugation at 16,000 × *g* for 10 minutes at 4°C, and the supernatant was collected.

For immunoprecipitation, 50 µL of Protein A/G beads were washed and equilibrated three times with IP lysis buffer. Subsequently, 100 µL of cell lysate and 10 µL of anti-αCat antibody were added to the beads and incubated overnight at 4°C with gentle rotation. The beads were then washed three times with IP lysis buffer. Immunoprecipitated proteins were eluted in 50 µL of 2× Laemmli buffer and samples were heated at 95°C for 15 minutes. Samples were stored at −20°C until Western blot analysis. Total protein concentration was quantified using the BCA Protein Assay Kit (Thermo Scientific), and 20 µg of total cell lysate was loaded as the input sample.

Gene Enrichment Analysis

Significantly deregulated phosphoproteins (FoldChange > 2, FDR < 0.1) following K^+ stimulation of NCI-H295R cells(1) were analyzed for molecular function Gene Ontology (GO) terms and Reactome pathway enrichment using EnrichR(3).

Cell Dissociation Assays

Cell dissociation assays were performed as described previously (4). Briefly, 60% confluent monolayers of NCI-H295R cells were treated with either CHIR (5 µM) or vehicle (DMSO) for 48 hours. Cells were detached using plastic cell scrapers and collected in 3 mL PBS. Cell suspensions were subjected to mechanical stress via repeated trituration (30 passes) using a plastic Pasteur pipette. Samples were fixed with 2.5% glutaraldehyde and 2.5% paraformaldehyde (PFA), then stained with phalloidin (1:1000) and Hoechst (1:1000), and visualized using a Nikon 90i microscope. The degree of cell dissociation was quantified by counting particles (single cells or cell clusters) and the total number of cells within each particle in seven different fields of view per replica. Data were expressed as the ratio nP/nC, where nP = number of particles and nC = total number of cells (a higher ratio indicates greater dissociation).

Cell Proliferation Assay and Nuclear Morphology Assessment

NCI-H295R cells (100,000 cells per well) were plated in 6-well plates and treated with fasudil (10, 50, and 100 µM) or vehicle (DMSO) for 10 days. Media were refreshed every 2–3 days with fresh fasudil or vehicle. At the

endpoint, cells were detached using 0.05% trypsin-EDTA and counted using a hemocytometer. Cell counts were normalized to the seeding number and expressed as fold change relative to seeding number.

For nuclear morphology assessment, cells from the same treatment conditions were trypsinized and replated onto poly-L-lysine–coated coverslips and cultured for one additional day. Cells were fixed with 4% PFA for 15 minutes, permeabilized with PBS-Triton X-100 (PBS-T) for 10 minutes, and stained with DAPI to visualize nuclei. Coverslips were mounted using ProLong Gold antifade mountant (Invitrogen) and imaged using a Zeiss LSM 700 confocal microscope.

Caspase 3/7 Luminescence Assay

NCI-H295R cells (8,000 cells per well) were plated in white-walled, opaque 96-well plates and cultured for 3 days to allow cell attachment and baseline equilibration. Cells were then pre-incubated with fasudil (10 or 100 μM) or vehicle (DMSO) for 1 hour, followed by treatment with CHIR (5 μM) or vehicle for 6 hours. At the endpoint, apoptosis was quantified using the Caspase-Glo 3/7 Assay System (Promega, G8091) according to the manufacturer's protocol. Luminescence was measured using a plate reader. Data are expressed as a percentage of the vehicle-treated control, with the average luminescence of the DMSO group set to 100%.

Primary Adrenal Cell Isolation and Culture

Adrenal glands from control and βCat-GOF mice were dissected, adherent adipose tissue was carefully removed and adrenal tissues were minced. Mince adrenals were pooled for each genotype and digested in 4mg/ml collagenase type I (Gibco, 17100-017) in PBS (250ul of the collagenase solution per adrenal) for 30 minutes at 37°C. Cell were then centrifuged and resuspended in DMEM-F12 supplemented with 10% fetal bovine serum (FBS), 1% ITS, 1% GlutaMAX, and 1% penicillin-streptomycin. Cells were plated into 48-well plates such that one adrenal corresponded to one well. After three days in culture, the medium was replaced with fresh medium and cells were incubated overnight. The following morning, the medium was replaced again, and cells were cultured for an additional 2 hours and then collected for aldosterone measurement. Cells were counted in each well, and aldosterone concentrations were normalized to cell number and expressed as fold change relative to control cells.

For staining experiment, primary adrenal cells were resuspended in 25 ul Matrigel (Corning) per well, and plated as domes in 24 well plate. Cells were plated as 2 adrenals per well. Upon the appearance of visible colonies, cultures were switched to fresh medium overnight. Matrigel domes were washed with PBS and fixed in 4% PFA on ice for 20 minutes with gentle shaking. Fixed domes were transferred to microfuge tubes,spinned-down, and washed three times with PBS. Cells were permeabilized with 0.3% Triton X-100 in PBS for 20 minutes with shaking, then blocked in 5% BSA in PBS for 1.5 hours. Samples were incubated with KCad primary antibody (1:100 in 1% BSA, 0.1% Triton X-100 in PBS) overnight at 4 °C. The following day, cells were washed three times with 0.1% Triton X-100 in PBS and incubated with fluorescent secondary antibody (1:400 in 1% BSA, 0.1% Triton X-100, in PBS) for 2 hours at room temperature. After three final washes in PBS, stained cells were mounted using ProLong Gold antifade mountant (Invitrogen) and imaged using a Zeiss LSM 700 confocal microscope.

References

1. Berber M, et al. Calcineurin regulates aldosterone production via dephosphorylation of NFATC4. *JCI Insight*. 2023;8(14).
2. Leng S, et al. β-Catenin and FGFR2 regulate postnatal rosette-based adrenocortical morphogenesis. *Nature Communications*. 2020;11(1):1–14.
3. Xie Z, et al. Gene Set Knowledge Discovery with Enrichr. *Curr Protoc*. 2021;1(3):e90.
4. Takeda H, et al. V-src kinase shifts the cadherin-based cell adhesion from the strong to the weak state and beta catenin is not required for the shift. *J Cell Biol*. 1995;131(6 Pt 2):1839–47.
