# Supplementary figures and images for: Rho/ROCK signaling and α-catenin mediate β-catenin–driven hyperplasia in the adrenal cortex via adherens junctions
Source: J Clin Invest. 2026 Jan 27;136(6):e196271. doi: 10.1172/JCI196271 (PMC12987613; doi:10.1172/JCI196271)

Full unedited blots

Panel Figure 1B

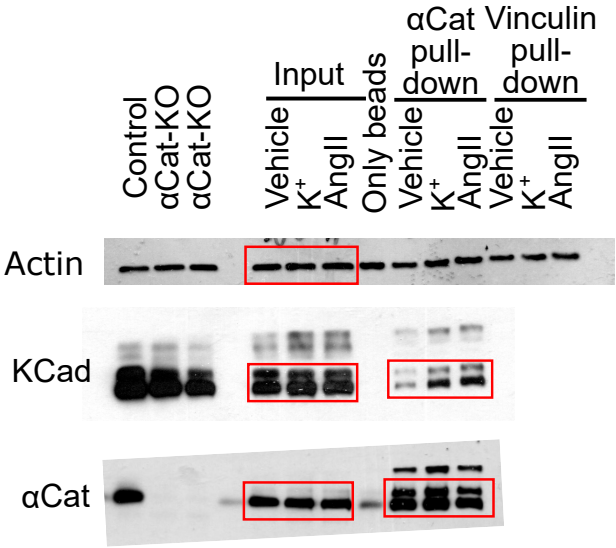

Panel Figure 1H

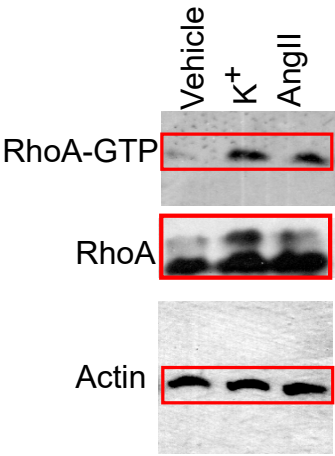

Panel Supplementary Figure 3F

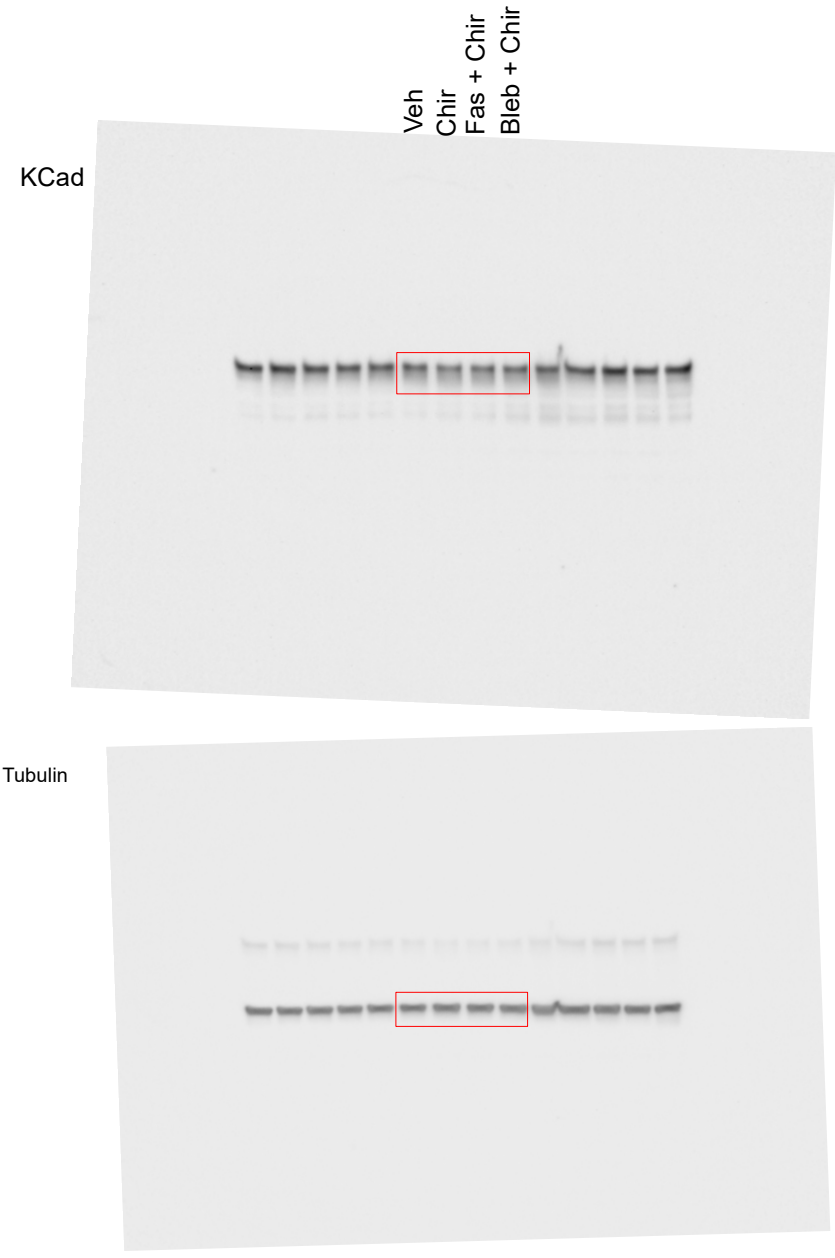

Supplement: Unedited blot and gel images [file jci-136-196271-s075.pdf]
